# Developing a machine learning algorithm to predict psychotropic drugs-induced weight gain and the effectiveness of anti-obesity drugs in patients with severe mental illness: Protocol for a prospective cohort study

**DOI:** 10.1371/journal.pone.0324000

**Published:** 2025-05-19

**Authors:** Hye Jun Lee, Na Yeon Kim, Da Seul Kim, Youngbin Kim, Jung-Ha Kim, Doug Hyun Han, Sun Mi Kim

**Affiliations:** 1 Department of Family Medicine, College of Medicine, Chung-Ang University, Seoul, Republic of Korea; 2 Biomedical Research Institute, Chung-Ang University Hospital, Seoul, Republic of Korea; 3 Department of Psychiatry, College of Medicine, Chung-Ang University, Seoul, Republic of Korea; 4 Department of Artificial Intelligence, Chung-Ang University, Seoul, Republic of Korea; University of Diyala College of Medicine, UNITED STATES OF AMERICA

## Abstract

Obesity is a global public health concern, often co-occurring in patients with severe mental illnesses. The impact of psychotropic drugs-induced weight gain is augmenting the disease burden and healthcare expenditure. However, predictors of psychotropic drug-induced weight gain and the efficacy of anti-obesity drugs remain underexplored. This study aims to develop a machine learning algorithm to predict both psychotropic drugs-induced weight gain and metabolic changes, and the potential of anti-obesity drugs. We plan to enroll 300 patients with severe mental illnesses, including schizophrenia, bipolar disorder, and major depressive disorder. In Phase 1, the study will predict weight gain and metabolic changes after the psychotropic treatment. Data on demographics, lifestyle, medical history, psychological factors, anthropometrics, and laboratory results will be collected at baseline and re-evaluated 24 weeks post-treatment. Participants classified as obese (body mass index ≥ 25 kg/m²) or overweight (body mass index of 23–24.9 kg/m²) at the 24-week follow-up will proceed to Phase 2, which focuses on predicting the promise of anti-obesity drugs. The study participants will receive anti-obesity medications for 24 weeks, and the same variables from Phase 1 will be reassessed. A machine learning model will be developed to predict both psychotropic drug-induced weight gain and anti-obesity medications that will be effective. The algorithm will be tailored to each patient to guide clinicians in personalizing psychiatric and obesity treatment plans. The clinical trial is registered with the Clinical Research Information Service, part of the WHO International Clinical Trials Registry Platform (approval number: KCT0009769).

## Introduction

Obesity is among the most important health problems, and the recent COVID-19 pandemic has increased the disease burden [1]. According to the World Health Organization (WHO, 2023), 43% of the world’s adult population was either obese or overweight in 2022, and the World Obesity Federation predicts that half the world’s population will turn obese by 2035 [2]. Obesity is defined as a body mass index (BMI) of 25 kg/m2 or more in Asia, and the prevalence of obesity among South Korean adults was 37.2% in 2022 [3]. The prevalence of obesity among people with severe mental illnesses (SMI) such as schizophrenia, bipolar disorder, and major depressive disorder is 25.9%; when overweight is included, the prevalence increases by more than half, reaching 60.1% worldwide [4]. Compared to the general population, people with SMI are 3.04 times more likely to become obese and 2.03 times more likely to be overweight (BMI of 23–24.9 kg/m²) or obese (BMI ≥ 25 kg/m²) [4].

Weight gain increases the risk of developing chronic diseases and associated complications or comorbidities, such as metabolic syndrome, including type 2 diabetes, hypertension, dyslipidemia, cardiovascular diseases, cancer, gastrointestinal and gallbladder diseases, musculoskeletal diseases [5]. Obesity increases the risk of developing social and mental disability and death, and it is associated with lower health-related quality of life and catastrophic health expenditure [6,7]. Among individuals with SMI, the mortality rate is two to three times higher than that of the general population, reducing life expectancy by 10–20 years—a gap that seems to be increasing [8]. About 60% of the excess mortality in this population group is attributed to physical comorbidities, primarily cardiovascular diseases [9].

Factors such as antipsychotic medications and unhealthy lifestyle habits contribute to the higher risk of weight gain in SMI [8]. Some psychotropic drugs (i.e., antipsychotics, antidepressants, and mood stabilizers) frequently cause weight gain and metabolic changes. Among the top-prescribed antipsychotics in South Korea, olanzapine and quetiapine are known to induce dose-dependent weight gain and affect various metabolic indicators [10,11]. Clinical experience shows that aripiprazole, initially perceived to have a lower risk of weight gain due to its mechanism of action, is associated with weight gain [11]. In a meta-analysis of weight gain as the side effect of antidepressants, mirtazapine showed the highest risk of weight gain, excluding tricyclic antidepressants that are no longer used as first-line therapy in the treatment of depression [10,12,13]. Furthermore, valproic acid, which is a commonly prescribed mood stabilizer, is associated with weight gain in up to 50% of patients [14]. Weight gain caused by psychotropic drugs is linked to an increased risk of metabolic syndrome, cardiovascular and cerebrovascular diseases, and high mortality rates [8]. Weight gain lowers self-esteem, reduces quality of life, increases healthcare costs, and affects treatment adherence due to weight gain as a side effect [15].

There is a significant gap in research regarding predictors of the side effects of weight gain associated with psychotropic drug treatment in patients with mental illnesses. Although individuals respond differently to the same psychotropic drugs, with notable variations in sensitivity to weight gain, studies exploring factors that could predict the degree of weight gain triggered by these medications in each mental health condition are limited. A combination of factors influence the risk of weight gain due to psychotropic medications, including genetic predispositions, lifestyle habits, body composition, and the severity of psychiatric symptoms [10,16] Comprehensive research must identify predictors of weight gain that encompass all these elements. Recent studies have applied machine learning techniques to predict obesity risk in the general adult population [17,18], but machine learning research to predict the risk of weight gain from psychotropic medications among individuals with mental illness is lacking.

Notwithstanding the widespread prescription of anti-obesity drugs, research on medications that would be most effective with fewer side effects remains limited. The current non-personalized approach to prescribing anti-obesity drugs may reduce treatment effectiveness, lead to the misuse of these medications, increase the risk of adverse effects, and result in unnecessary healthcare expenditures [19,20]. Multiple factors determine the efficacy and side effects of anti-obesity medications, including genetic background, socioeconomic status, lifestyle habits, body composition, comorbidities, and concurrent medication use [19,20]. There is a need for studies to explore the predictive factors of treatment effectiveness, considering all these variables.

This study aims to develop a machine learning algorithm to predict the likelihood of weight gain and the metabolic side effects associated with the use of psychotropic drugs. This machine learning algorithm will also determine the anti-obesity drugs that are most effective in managing weight gain related to the use of psychotropic drugs. This study presents the protocol for a prospective cohort study designed to develop these machine learning algorithms.

### Objectives

Develop a machine learning algorithm (1) to predict weight gain and the metabolic changes induced by psychotropic drugs (Phase 1); and (2) to predict the efficacy of anti-obesity drugs in obese or pre-obese patients with weight gain associated with psychotropic drugs (Phase 2).

## Materials and methods

### Study design

Develop machine learning algorithms based on data collected in a prospective cohort study.

### Recruitment

**Fig 1:**
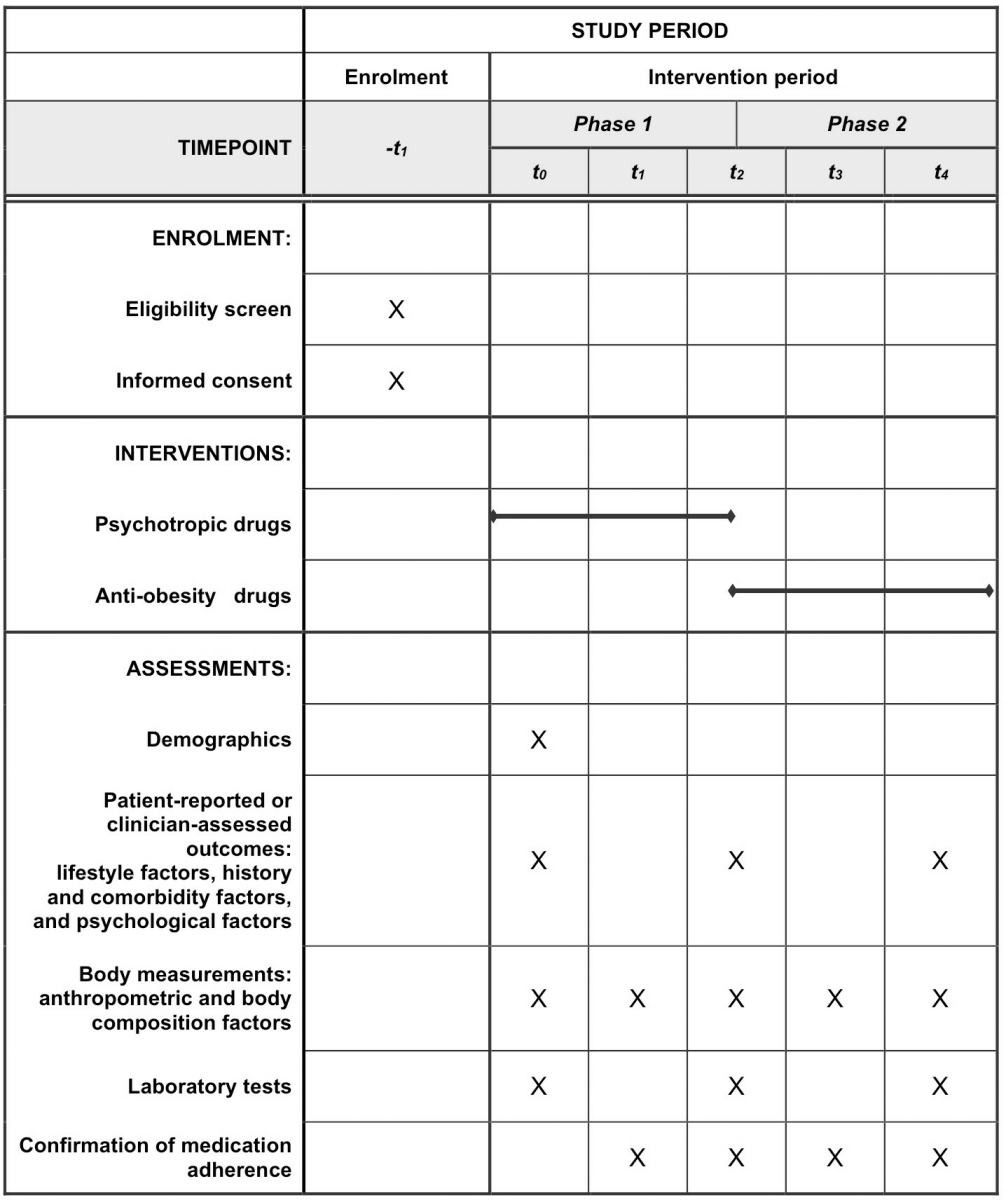
Schedule of enrolment, interventions, and assessments. Note: *-t*_*1*_: -2 weeks; *t*_*0*_: baseline; *t*_*1*_: 12 weeks, *t*_*2*_: 24 weeks; *t*_*3*_: 36 weeks; *t*_*4*_: 48 weeks. *Phase 1*: Study predicting psychotropic drug-induced weight gain. *Phase 2*: Study assessing the efficacy of anti-obesity drugs in managing psychotropic-drug induced weight gain. A total of 300 participants will be enrolled. Participants with a BMI ≥ 25 kg/m² (obese) or 23–24.9 kg/m² (overweight) at the end of Phase 1 will be enrolled in Phase 2.

Participants will be recruited by posting a notice at the Department of Psychiatry, Chung-Ang University Hospital, and the study information sheets will be distributed to both outpatients and inpatients. Participants who volunteer will be screened to determine if they meet the inclusion and exclusion criteria before enrollment. We aim to enroll a total of 300 participants. Participants with a BMI ≥ 25 kg/m² (obese) or 23–24.9 kg/m² (overweight) at the end of Phase 1 will be enrolled in Phase 2 (Fig 1). The study protocol (version 1.5) was approved by the Institutional Review Board (IRB) of the Chung-Ang University Hospital (approval number: 2406-013-603). Prior to participation, the study participants will provide informed consent, and they will be evaluated to determine their ability to provide informed consent following ethical guidelines and institutional requirements. The participants will be assessed to confirm their understanding of the study purpose, procedures, risks, benefits, and their right to withdraw. This evaluation process will explain the study in simple terms and confirm understanding through open-ended questions. Participants will be deemed capable if they demonstrate comprehension, that is, the ability to communicate their decision and consistent decision-making that reflects their values. The Chung-Ang University Hospital’s IRB has approved the consent procedure and assessment and data analysis methods to ensure adherence to the ethical standards set by the Korean Association of IRBs and the Helsinki Declaration, with anonymization and ongoing ethics training for team members. Adverse events will be documented and reported in compliance with the guidelines of Chung-Ang University Hospital’s IRB. Participants will be discontinued if they explicitly express their intent to withdraw, fail to adhere to the prescribed medication for more than two weeks without consulting the medical team, or if clinical findings emerge during the trial that necessitate their exclusion. Modifications to the study protocol will require IRB approval before implementation in the trial. The clinical trial was registered with the Clinical Research Information Service, part of the WHO International Clinical Trials Registry Platform, on September 4, 2024 (approval number: KCT0009769; https://cris.nih.go.kr/cris/search/detailSearch.do?seq=27988&search_page=L). Recruitment process Participants has been ongoing since September 9, 2024 and the process will continue until March 31, 2027. Data collection will conclude by September 30, 2027, and the results are expected by December 2027.

### Eligibility criteria

#### Phase 1: Study on predicting psychotropic drug-induced weight gain.

The inclusion criteria are: (1) aged 19 years or older; (2) diagnosed with major depressive disorder, bipolar disorder, or schizophrenia according to the Diagnostic and Statistical Manual of Mental Disorders, Fifth Edition (DSM-5) diagnostic criteria [21]; (3) having no history of olanzapine, quetiapine, aripiprazole, mirtazapine, or valproate within one month of initial evaluation; and (4) having no history of using anti-obesity drugs for weight loss within one month from the time of initial evaluation. The exclusion criteria are: (1) being diagnosed with a severe medical illness (malignancy; severe heart disease such as heart failure; chronic kidney disease; severe hepatic disease; severe pulmonary disease; and pancreatic diseases such as pancreatitis, thyroid disease, and glaucoma); (2) having a history of past or present substance dependence or abuse; and (3) history of epilepsy, head trauma, or organic mental disorder.

#### Phase 2: Study on predicting the efficacy of anti-obesity drugs in cases of psychotropic-induced weight gain.

Participants who complete Phase 1 and have a BMI of 25 kg/m² or higher (classified as obese according to the WHO Asia-Pacific criteria [22]) or a BMI between 23 kg/m² and 25 kg/m² (classified as overweight or pre-obese according to the WHO Asia-Pacific criteria [22]) at the end of the Phase 1 will be included. The exclusion criteria will be the same as those in Phase 1.

### Interventions and measures

#### Phase 1.

Demographic factors, lifestyle factors, history and comorbidity factors, anthropometric and body composition factors, and laboratory data will be measured at baseline (Table 1). If the participant is already taking psychotropic medications, the current medication will be maintained, and one of the following medications, known to have significant weight gain side effects, will be additionally administered for 24 weeks: olanzapine, quetiapine, aripiprazole, mirtazapine, or valproic acid (Table 2). Anthropometric and body composition factors will be measured 12 weeks after the initiation of the psychotropic medication. All other factors, except demographic factors, will be reassessed at the end of the 24-week period. During Phase 1, participants must abstain from using anti-obesity medications for weight loss.

**Table 1:** Summary of data collection schedule during phase 1.

Schedule				Screening	T0	T1	T2
					Baseline	12 weeks	24 weeks
**Screening**		Inclusion/exclusion criteria		×			
		Written informed consent		×			
**Intervention**		Taking psychotropic drugs; olanzapine, quetiapine, aripiprazole, mirtazapine, valproate					
**Evaluation**	**Demographics**	Demographic factors			×		
	**Patient-reported or clinician-assessed outcomes**	Lifestyle factors	Total daily energy and nutrient intake (24-hour recall method)		×		×
			Eating attitude (KEAT-26)		×		×
			Physical activity (IPAQ)		×		×
			Alcohol intake (AUDIT-K)		×		×
			Sleep (ISI-K)		×		×
		History and comorbidity factors	Family history of obesity		×		
			Comorbidities (including metabolic syndrome)		×		
			History of taking anti-obesity drug		×		
		Psychological factors	Symptoms of schizophrenia (PANSS)		×		×
			Depressive symptoms (BDI-II)		×		×
			Bipolar symptoms (MDQ)		×		×
	**Body measurements**	Anthropometric and body composition factors	Height-to- waist circumference, waist-to-hip circumference ratio, and weight		×	×	×
			Body mass index, body fat mass, lean body mass, skeletal muscle mass, abdominal fat percentage, and basal metabolic rate (bioelectrical impedance analysis)		×	×	×
	**Laboratory tests**	Insulin, fasting blood sugar, lipids (total cholesterol, low-density lipoprotein cholesterol, high-density lipoprotein cholesterol, triglycerides), uric acid, and high-sensitivity C-reactive protein			×		×
**Confirmation**	**Medication**	**Medication adherence**				×	×

Note: KEAT-26, the Korean version of Eating Attitudes Test-26; IPAQ, the Korean version of short-term International Physical Activity Questionnaire; AUDIT-K, the Korean version of Alcohol Use Disorder Identification Test; ISI-K, the Korean version of the Insomnia Severity Index; PANSS, Positive and Negative Syndrome Scale; BDI-II, Beck Depression Inventory-II; MDQ, Mood Disorder Questionnaire.

**Table 2:** Psychotropic drugs known to cause significant weight gain as a side effect, and metabolic side effects among the most prescribed in South Korea.

Ingredient name	Mechanism	Usage	Indication	Contraindication
Olanzapine [23]	Atypical antipsychotics	Initial 5–10 mg once daily, usual dosage range is 10–20 mg/day	Schizophrenia, Bipolar disorder	Patients with angle-closure glaucoma or unstable medical condition
Quetiapine [23]	Atypical antipsychotics	Initial 25 mg/day twice a day, usual dosage range is 400–800 mg/day	Schizophrenia, Bipolar disorder	Patients with a proven allergy to quetiapine
Aripiprazole [23]	Atypical antipsychotics	Initial dose is 10–15 mg once daily, usual dosage range is 15–30 mg/day	Schizophrenia, Bipolar disorder, Major depressive disorder	Patients with a proven allergy to aripiprazole
Mirtazapine [24]	Anti-depressant	The initial recommended dose is 15 mg once a day; the dose is increased to a maximum of 45 mg/day	Major depressive disorder	Patients with hypersensitivity to the components of this drug or patients receiving MAO (monoamine oxidase inhibitors) inhibitors
Valproate [24]	Antipsychotic, Anti-convulsant	The initial recommended dose is 20 mg per kg of body weight, divided and orally administered 1–2 times a day, and the recommended maintenance dose is 1,000–2,000 mg per day	Bipolar disorder	Patients with hypersensitivity to the components of this drug, pregnant women. or women who may become pregnant, (severe) liver disease, severe pancreatic disease, urea cycle disease, or patients taking carbapenem antibiotics, patients with porphyria, patients receiving mefloquine, patients with systemic primary carnitine deficiency with uncorrected hypocarnitinemia

#### Phase 2.

If the participants are obese or pre-obese (overweight) at the time of the follow-up assessment (week 24) during Phase 1, they will progress to Phase 2 (Table 3). Anti-obesity drugs will be used among the currently approved drugs (orlistat, naltrexone/bupropion, liraglutide, semaglutide, phentermine/topiramate, and phentermine), metformin, or topiramate prescribed for weight loss (Table 4). It is prescribed based on the physician’s judgment and the patient’s baseline condition. However, as phentermine is currently approved in South Korea as a short-term appetite suppressant (up to 12 weeks of use), anthropometric measurements and body composition factors will be measured after 4 weeks, administration will be discontinued within 12 weeks, and a follow-up evaluation will be conducted. Subsequently, data will be collected by re-measuring the same factors as in Phase 1 after administering anti-obesity drugs for 24 weeks in obese or overweight patients.

**Table 3:** Summary of data collection schedule for phase 2.

Schedule				Screening	T0	T1	T2
					Baseline	12 weeks	24 weeks
Screening		Inclusion/exclusion criteria		×			
		Written informed consent		×			
Intervention		Taking anti-obesity drugs: orlistat, naltrexone/bupropion, liraglutide, semaglutide, phentermine/topiramate, phentermine, metformin, or topiramate					
Evaluation	Demographics	Demographic factors			×		
	Patient-reported or clinician-assessed outcomes	Lifestyle factors	Total daily energy and nutrient intake (24-hour recall method)		×		×
			Eating attitude (KEAT-26)		×		×
			Physical activity (IPAQ, 7)		×		×
			Alcohol intake (AUDIT-K)		×		×
			Sleep (ISI-K)		×		×
		History and comorbidity factors	Family history of obesity		×		
			Comorbidities (including metabolic syndrome)		×		
			History of taking anti-obesity drug		×		
	Body measurements	Anthropometric and body composition factors	Height-to- waist circumference; waist-to-hip circumference ratio, and weight		×	×	×
			Body mass index, body fat mass, lean body mass, skeletal muscle mass, abdominal fat percentage, and basal metabolic rate (bioelectric impedance analysis)		×	×	×
	Laboratory tests	Insulin, fasting blood sugar, lipids (total cholesterol, low-density lipoprotein cholesterol, high-density lipoprotein cholesterol, triglycerides), uric acid, and high-sensitivity C-reactive protein			×		×
Confirmation	Medication	Medication adherence				×	×

Note: KEAT-26, the Korean version of Eating Attitudes Test-26; IPAQ, the Korean version of short-term International Physical Activity Questionnaire; AUDIT-K, the Korean version of Alcohol Use Disorder Identification Test; ISI-K, the Korean version of the Insomnia Severity Index.

**Table 4:** Anti-obesity drugs used in this study.

Ingredient name	Mechanism	Usage	Indication	Contraindication
Orlistat [25]	Gastric/pancreatic lipase inhibitor	Administered orally 60–120 mg once 3 times a day	Obese patients with a BMI of 30 kg/m² or more, or 27 kg/m² or more but less than 30 kg/m^2^ with other risk factors (e.g., hypertension, diabetes, dyslipidemia)	Patients with chronic malabsorption syndrome or cholestasis; pregnant women, or women who may become pregnant, and lactating mothers
Naltrexone/bupropion [26]	Opioid receptor antagonist	The starting dose is administered once a day, 1 tablet per time, and the dose is increased over 4 weeks	Obese patients with a BMI of 30 kg/m² or more or 27 kg/m² or more but less than 30 kg/m^2^ with other risk factors (e.g., hypertension, diabetes, dyslipidemia)	Patients with uncontrolled high blood pressure, seizure disorder or history of seizures, central nervous system tumor, sudden discontinuation of alcohol or drugs such as benzodiazepines, barbiturates, antiepileptic drugs, bipolar disorder, or other drugs containing bupropion or naltrexone. Patients with a current or past diagnosis of bulimia or anorexia nervosa, current dependence on opiates or opiate agonists (e.g., methadone), or patients with acute opiate withdrawal symptoms. Patients taking MAO inhibitors, patients with acute hepatitis or liver failure, severe liver failure, end-stage renal disease, pregnant women, women who may become pregnant, lactating mothers, and those aged 75 years or above.
Phentermine/topiramate [27]	Sympathomimetic amine	Take the initial dose of 3.75 mg/2 3 mg daily for 14 days. After 14 days, take the recommended dose of 7.5 mg/46 mg. (If necessary, escalate to 11.25 mg/69 mg, 15 mg/92 mg)	Obese patients with a BMI of 30 kg/m² or more, or 27 kg/m² or more but less than 30 kg/m^2^ with other risk factors (e.g., hypertension, diabetes, dyslipidemia)	Pregnant women, glaucoma, hyperthyroidism, patients taking MAO inhibitors or less than 14 days after taking them, patients with hypersensitivity to sympathomimetic amines or idiosyncratic constitution, worsened arteriosclerosis, cardiovascular disease, moderate to severe hypertension, pulmonary hypertension, mental anxiety or excitement, history of drug abuse
Liragluti-de [28]	GLP-1 receptor agonist	The starting dose is 0.6 mg once a day, and it is gradually increased by 0.6 mg at intervals of 1 week or more to a maintenance dose of 3.0 mg once a day via subcutaneous injection.	Obese patients with a BMI of 30 kg/m² or more, or 27 kg/m² or more but less than 30 kg/m^2^ with other risk factors (e.g., hypertension, diabetes, dyslipidemia)	Patients with past medical history or family history of medullary thyroid carcinoma or multiple endocrine neoplasia syndrome type 2
Semaglutide [28]	GLP-1 receptor agonist	The starting dose is 0.25 mg per week, with gradual increases to 0.5 mg, 1.0 mg, 1.7 mg, and 2.4 mg, with intervals of 1 month or longer, reaching a maintenance dose of 2.4 mg per week through subcutaneous injection.	Obese patients with a BMI of 30 kg/m² or higher, or those with a BMI of 27 kg/m² or higher but less than 30 kg/m^2^ and have other risk factors (e.g., hypertension, diabetes, or dyslipidemia)	Patients with a past medical history or family history of medullary thyroid carcinoma or multiple endocrine neoplasia type 2
Phentermine [29]	Sympathomimetic amine	15–37.5 mg is given orally once daily, administered for a short period of time (within 4 weeks, and administered for up to 12 weeks)	Obese patients with a BMI of 30 kg/m² or more or 27 kg/m² or more but less than 30 kg/m^2^ with other risk factors (e.g., hypertension, diabetes, dyslipidemia)	Pregnant women, glaucoma, hyperthyroidism, patients taking MAO inhibitors or less than 14 days after taking them, patients with hypersensitivity to sympathomimetic amines or idiosyncratic constitution, worsened arteriosclerosis, cardiovascular disease, moderate to severe hypertension, pulmonary hypertension, mental anxiety or excitement, history of drug abuse
Metformin [30]	Hypoglycemic agent	Initial dose is 500 mg once a day (maximum 2000 mg per day)	Metformin is not FDA-approved for weight loss but is effective in obese or overweight diabetic patients.	Patients with moderate (Stage 3b) and severe renal disease (creatinine clearance <45 ml/min or glomerular filtration rate <45 ml/ min/1.73 m^2^), acute conditions that may affect renal function such as dehydration, serious infection, cardiovascular collapse (shock), acute myocardial infarction, sepsis, acute and unstable heart failure, type 1 diabetes, lactic acidosis, patients with acute or chronic metabolic acidosis, including diabetic ketoacidosis with or without coma, patients with a history of ketoacidosis, diabetic precoma, malnutrition, starvation, weakness, pituitary insufficiency, or adrenal insufficiency. Acute or chronic diseases that can cause tissue hypoxia such as hepatic dysfunction, respiratory failure, acute myocardial infarction and shock, excessive alcohol intake, and patients with gastrointestinal disorders such as dehydration, diarrhea, and vomiting.
Topiramate [31,32]	Anti-convulsant	Start with an initial dose of 25 mg once a day, then increase the dose by 25 or 50 mg per day at 1–2-week intervals (recommended dose is 100–200 mg per day).	Topiramate is not FDA-approved for weight loss but is used off-label for obesity, alcohol use disorder, binge eating, and bulimia nervosa	Patients who currently have or are at risk for metabolic acidosis. Patients with hypersensitivity to the ingredients of this drug.

Note: BMI, body mass index; GLP, glucagon-like peptide; MAO, monoamine oxidase

#### Measurements.

Alongside demographic factors, the following lifestyle factors will be measured: (1) total daily energy and nutrient intake using the 24-hour recall method; (2) eating attitudes using the Korean version of the Eating Attitudes Test-26 (KEAT-26) [33]; (3) physical activity using the Korean version of the Short-term International Physical Activity Questionnaire (IPAQ) [34]; (4) alcohol abuse by assessing the Korean version of Alcohol Use Disorder Identification Test (AUDIT-K) [35]; and (5) sleep problems using the Korean version of the Insomnia Severity Index (ISI-K) [36].

Family history and comorbidity factors including family history of obesity, comorbid medical problems (including metabolic syndrome), and history of taking prescription anti-obesity drug will be assessed. Anthropometric measurements and body composition factors will be measured using bioelectrical impedance analysis: height, waist circumference, waist/hip circumference ratio, weight, BMI, body fat mass, lean body mass, skeletal muscle mass, abdominal fat percentage, and basal metabolic rate.

The following psychological factors will be also measured: (1) positive and negative symptoms of schizophrenia through structured interviews using the Positive and Negative Syndrome Scale (PANSS; 30 questions) [37]; (2) level of depressive symptoms using the Beck Depression Inventory-II (BDI-II, 21 questions) [38,39]; and (3) symptoms of bipolar spectrum disorder using the Mood Disorder Questionnaire (MDQ, 15 questions) [40].

Laboratory tests will be conducted to measure insulin, fasting blood sugar, lipids (total cholesterol, low-density lipoprotein cholesterol, high-density lipoprotein cholesterol, and triglycerides), uric acid, and high-sensitivity C-reactive protein. In Phase 2, if necessary, electrocardiograms and pregnancy tests will be conducted to check for contraindications before prescribing anti-obesity drugs.

### Development process of deep learning model

Using the data collected, we propose to develop a deep learning model that recognizes the changes induced by a psychotropic drug in weight-, obesity-, and metabolism-related factors (Phase 1). We will also develop machine learning algorithms that examine through deep learning the amount of change that each anti-obesity drug causes in body weight-, obesity-, and metabolism-related factors in obese or pre-obese patients who gained weight from using psychotropic drugs (Phase 2). The development processes of the deep learning models are as follows.

#### Data augmentation.

We will apply data augmentation techniques to handle small datasets. SimSiam, a contrastive learning method, will be used to generate augmented views of the data by applying weights and enhancing model learning from different perspectives [41]. A variational autoencoder will be used to artificially expand the tabular dataset by generating synthetic data that reflect the statistical properties of the original data [42]. These methods aim to improve the model’s robustness and performance with limited data.

#### Deep learning-based model design.

In the planned design of our deep learning-based model, we intend to incorporate methodologies that effectively address the complexities inherent in clinical and biological data. A hybrid architecture combining tree-based [43] and transformer-based [44] models will be employed, specifically TabNet [45] and TabLLM [46], given their proven strengths in capturing complex, non-linear interactions and handling structured medical datasets. Advanced representation learning approaches, including self-supervised learning and graph neural networks [47], will be explored to further improve the proposed model’s ability to capture the subtle relationships between clinical, psychological, anthropometric, and metabolic factors.

Considering the characteristics of deep learning models, explicit feature selection will not be separately performed. Instead, post-hoc interpretability methods, such as SHapley Additive exPlanations (SHAP), will be applied to assess the relative importance and clinical relevance of individual features after model training.

For model validation, data will be split into training (~70%), validation (~15%), and independent test (~15%) sets. Computationally, model training and evaluation will be conducted using NVIDIA A100 GPUs (40GB VRAM) with PyTorch as the primary deep learning framework. Given our dataset size and model complexity, we anticipate a total training duration of approximately 12–24 hours per model configuration.

#### Deep-learning-based model training and evaluation.

Key techniques will be implemented in the deep learning-based model training and evaluation phase. An attention mechanism [48] will be applied to capture the time series characteristics of each factor, ensuring that temporal dependencies are properly accounted for. To enhance model generalization and data efficiency, the model-agnostic meta-learning approach will be employed [49]. The model’s performance will be assessed using various metrics, including the root mean squared error for predicting changes in factors, the area under the receiver operating characteristic curve and precision-recall curves for classification tasks, and specialized metrics for medical predictions.

Advanced hyperparameter optimization techniques, such as the Bayesian optimization method [50], will be used to adjust the model for optimal performance. Model interpretability will be emphasized using integrating techniques such as the SHapley Additive ExPlanations values [51] and Local Interpretable Model-Agnostic Explanations [52] to clarify the model decisions beyond the traditional tree-based methods. These strategies aim to ensure a robust, interpretable, and high-performing model.

#### Ethical considerations and data privacy.

Ethical considerations and data privacy are prioritized in our deep learning model. To prevent bias, the fairness-aware machine learning techniques will be adopted. For privacy protection, federated learning [53] will be incorporated, enabling collaborative model training without sharing raw data across institutions, and applied differential privacy [54] to ensure that individual data remain secure by preventing any personal information from being inferred from the model’s outputs.

### Outcomes

The following study outcomes are expected: (1) a machine learning algorithm to predict weight gain and metabolic changes caused by psychotropic drugs (Phase 1), and (2) machine learning algorithms to predict the efficacy of anti-obesity drugs in obese or pre-obese patients with psychotropic drug-induced weight gain (Phase 2).

### Sample size

To develop a personalized pharmacotherapy model for patients with bipolar disorder using machine learning and deep learning techniques, Zheng et al. [55] used 164 final data points extracted from electronic medical records, reporting an accuracy range of 73% to 85%. Eder et al. [56] applied machine learning technology to predict the risk of weight gain associated with antipsychotic medications, antidepressants, and mood stabilizers, reporting an average accuracy of 66.1% to 79.2%. Initially, this study included 163 patients, but a high dropout rate of 37% after four weeks resulted in confirmed weight change data for only 103 participants.

The required sample size was estimated using G*Power 3.1.9.7 (Franz Faul, Kiel University, Germany, 2020), applying the “Linear multiple regression: Fixed model, R² increase” function. The calculation was based on a moderate effect size (*f*² = 0.10), a significance level (α) of 0.05, a statistical power (1 − β) of 0.80, and 10 predictor variables. Based on these parameters, the minimum required sample size was determined to be approximately 180 participants. Given an expected 40% dropout rate, the final target sample size was approximately 300 participants. This adjustment ensures sufficient statistical power while accounting for potential attrition during the study period.

We estimate about 180 participants to complete Phase 1 of the study. By the follow-up evaluation for Phase 1 (24 weeks), participants with a BMI of 25 kg/m² or higher (classified as obese) or a BMI between 23 kg/m² and 25 kg/m² (classified as overweight) will transition to Phase 2. As the prevalence of obesity and being overweight among individuals with SMI is 60.1% [4], approximately 110 participants will likely proceed to Phase 2, providing a sufficient sample size for developing the machine learning algorithm.

Considering the characteristics of psychiatric patient studies, where high dropout rates are expected [57], we plan to actively utilize interim data collected for up to three months for our analysis. Furthermore, multiple imputation methods and machine learning-based missing data handling techniques (e.g., autoencoders) will be applied to minimize data loss and enhance model performance. Applying these measures, we aim to maintain the reliability of our study results notwithstanding the anticipated high dropout rate.

### Data collection, management, quality control, and standardization

Qualified members of the research team, with expertise in family medicine, psychiatry, psychology, nursing, and experienced in handling obese or psychiatric patients, will be responsible for data collection and management. Machine learning-driven data management protocols will be integrated alongside medical expertise to ensure data accuracy and reliability.

To maintain standardized data collection, a structured protocol will ensure consistency across researchers and time points. Electronic data capture systems will be used to minimize errors and enable real-time monitoring. During the study period, the research team will undergo regular training to maintain consistency and quality control.

#### Missing data handling.

To effectively address missing data, multiple imputations will be used for statistical analyses, while predictive mean matching will be applied to numerical variables to replace missing values with realistic estimates. Machine learning applications, such as autoencoders, will be used to enhance model robustness.

#### Quality control and data management.

To ensure data accuracy and integrity, the research team will conduct regular data review meetings to identify inconsistencies and correct errors. Simplified data entry protocols will limit inputs to essential variables, incorporate drop-down menus for categorical data, and automate unit conversions to prevent manual errors. Automated validation rules will flag incorrect values, highlight missing fields, and prevent duplicate entries, thereby reducing the need for manual corrections.

#### Standardization and inter-rater reliability.

The study personnel will receive standardized training and will participate in regular calibration sessions to ensure consistent data collection. Inter-rater reliability will be assessed using the Intraclass Correlation Coefficient for continuous variables and Cohen’s Kappa for categorical variables to ensure accuracy across different raters.

#### Data security and integration.

All collected data will be stored in databases that are compliant with the Health Insurance Portability and Accountability Act with encryption, strict access controls, and activity logging to safeguard patient privacy. Version control systems will track modifications, while metadata documentation will ensure data traceability and reproducibility. Harmonization techniques will be applied to integrate diverse data sources, including questionnaires, laboratory tests, and body composition measurements. Longitudinal data tracking will ensure data completeness over multiple visits. Finally, de-identified datasets will be prepared for future research, subject to appropriate approvals.

By implementing these systematic approaches to data collection, quality control, standardization, and security, this study aims to ensure high-quality, accurate, and reproducible findings, while maintaining ethical and regulatory compliance.

### Considerations for medication changes

Nonadherence to the study protocol, including medication intake or follow-up assessments, may lead to participant dropout. However, there is a possibility that medications may need to be changed in patients with severe mental illness due to a deterioration of symptoms, which could impact study retention. In this study, if a patient is unable to complete the full six-month period in each phase owing to necessary medication change, such patients will be excluded from the six-month follow-up analysis. However, if interim data are available at the three-month mark, we plan to utilize such data.

Additionally, the potential to introduce new medications during the study period is likely. For example, if a new anti-obesity drug with significant efficacy is developed and becomes widely prescribed, we will seek IRB approval for protocol modification and include the new drug in our study.

### Interim analysis

Given the long-term potential for clinical research, this study includes a pilot study, interim analysis, and model deployment strategy. As part of the pilot study, an initial feasibility assessment will be conducted with 50 participants, allowing for necessary adjustments to the research protocol and methodology. Additionally, interim analysis will be performed at 12 months to evaluate the recruitment and dropout rates, ensuring that the study design can be modified if required.

## Results and discussion

Obesity has become a global epidemic, and people with SMI reportedly have a high incidence of obesity and associated complications. Rather than standardized treatment, patient-centered individualized care and the prevention of complications are important for optimizing patient outcomes. For example, the extent of weight gain induced by antipsychotic medication can vary depending on genetic differences between patients [58–60]. Individualized treatment may be emphasized to improve effectiveness and prevent side effects. When antipsychotic-induced metabolic disorders lead to obesity, medications such as metformin, liraglutide, and semaglutide are used to effectively correct metabolic disorders [28,61]. If psychotropic medications increase appetite levels, leading to obesity, appetite suppressants such as phentermine/topiramate or naltrexone/bupropion may be considered [62,63]. Tools that can predict the side effects of psychotropic medication in an individual and other tools that can predict the effectiveness of anti-obesity medication treatment are required; however, these tools are not yet available.

When machine learning technology becomes widely applicable, research on machine learning algorithms that address the issue of weight gain in patients with mental illness is expected to progress aggressively. A recent study involving 103 psychiatric inpatients employed a machine learning approach to predict a weight gain of ≥5% of body weight during the first four weeks of treatment with psychotropic drugs that are known to cause weight gain [56]. The key risk factors were identified as baseline BMI, premorbid BMI, age, eating behavior, and blood glucose levels. However, the study will likely have several limitations, including a limited range of independent variables, only inpatients, a short follow-up period, and difficulty in comparing weight gain across different drugs. More importantly, the earlier study did not develop a model to predict obesity treatment effectiveness—a key distinction from our study.

Unlike prior research, this study provides a comprehensive framework for personalized psychiatric care as it not only predicts psychotropic-induced weight gain but also offers individualized predictions for anti-obesity drug effectiveness.

To achieve this, we aim to develop a machine learning algorithm using deep learning techniques to calculate the likelihood of weight gain and metabolic side effects induced by psychotropic medications and identify the most effective anti-obesity drugs for individuals who experience weight gain due to psychotropic treatment. The algorithm will integrate a wide range of demographic, lifestyle, body composition, laboratory, and psychological data, allowing for a holistic assessment of the impact of different treatments on weight and metabolic health in psychiatric patients.

By incorporating predictive insights into the efficacy of anti-obesity drugs, the algorithm will assist clinicians to make more informed treatment decisions and develop personalized treatment plans that address obesity and the underlying psychiatric conditions. This approach is expected to improve patient outcomes, as it will reduce the burden of obesity-related health complications and optimize treatment strategies for patients with SMI.

The final machine learning model will be developed into a Clinical Decision Support System (CDSS) for real-world clinical application. This CDSS will enable clinicians to optimize treatment decisions by integrating predictive insights into routine psychiatric and metabolic care. Cost-effectiveness will be assessed in the future using healthcare utilization data. Through this translational approach, the study aims to establish a data-driven, personalized treatment framework for managing obesity in psychiatric patients, specifically those experiencing psychotropic-induced weight gain.

### Limitations

Despite the strengths, this study has several limitations, specifically the generalizability of findings and the potential overestimation of predictive accuracy in machine learning models. First, with an anticipated dropout rate of 40% in Phase I, participants who complete the full 24-week period may not be a random subset of the originally enrolled 300 participants. This introduces selection bias, as those who remain in the study may differ systematically from the broader psychiatric population in unmeasured or unidentified ways, limiting the external validity of the proposed model. Second, the machine learning models will be trained on observed data, which may lead to overestimation of prediction accuracy, particularly when applied to external populations or altered clinical settings. Without independent external validation, the proposed model’s generalizability remains uncertain. To address these limitations, we will conduct sensitivity analysis to assess the impact of dropout and perform external validation using independent datasets.

## Conclusion

This study aims to develop a machine learning algorithm that can predict weight change, metabolic changes, and the effectiveness of anti-obesity medications in psychotropic drugs-induced weight gain. This algorithm can be individually tailored for each patient to help select the optimal medication when establishing a psychiatric treatment plan and/or obesity treatment plan for patients with SMI.

## Supporting information

S1 File SPIRIT Checklist. (DOC)

S2 File Ethics Approval Letter. (PDF)

S3 File Study Protocol. (PDF)
