# Supplementary material for: Developing a machine learning algorithm to predict psychotropic drugs-induced weight gain and the effectiveness of anti-obesity drugs in patients with severe mental illness: Protocol for a prospective cohort study
Source: PLoS One. 2025 May 19;20(5):e0324000. doi: 10.1371/journal.pone.0324000 (PMC12088068; doi:10.1371/journal.pone.0324000)
Supplement: S2 File — (PDF) [file pone.0324000.s002.pdf]

Predicting the likelihood of weight gain as a side effect of psychiatric medications and the effectiveness of anti-obesity drugs: A pilot study

Version No 1.5

Affiliation of Principal Investigator: Department of Psychiatry
Principal Investigator: Sun Mi Kim

Research overview

Study Title	(English) Predicting the likelihood of weight gain as a side effect of psychiatric medications and the effectiveness of anti-obesity drugs: A pilot study
Principal Investigator	Sun Mi Kim, Department of Psychiatry
Funding Organization	Chung-Ang University

Research objectives	To develop an artificial intelligence algorithm to predict the occurrence of weight gain and metabolic side effects caused by psychotropic medications in individuals and to identify which anti-obesity medications would be effective in cases of psychotripic medication-induced obesity.
Study design	Longitudinal research studies
Study period	IRB approval date to 36 months
What to study (drugs, etc.)	(1) Psychotropic drugs: Olanzapine, quetiapine, aripiprazole, mirtazapine, and valproate.<br>(2) Obesity drugs: orlistat, naltrexone/bupropion, liraglutide, phentermine/topiramate, phentermine, semaglutide, and metformin (prescribed for weight control), and topiramate (prescribed for weight control).
Number of study Participants	300 participants
Vulnerable Populations	N/A
Research methods	**[Phase 1] Study on the potential for weight gain side effects of psychotropic medications**<br>Patients diagnosed with depression, bipolar disorder, or schizophrenia will be recruited. At the baseline evaluation, demographic factos, lifestyle factors, family history, comorbidities, anthropometric and body composition factors, hematologic factos, and psychological scales will be assessed. Based on the clinician's judgement, one of the following medications—olanzapine, quetiapine, aripiprazole, mirtazapine, or valproic acid— will be administered for 24 weeks. Anthropometric and body composition factors will be measured

	at 12 weeks, and all factors, except demographic factors, will be reassessed at 24 weeks.<br>**[Phase 2] Study on predicting the effectiveness of anti-obesity drugs in psychotropic drug-induced obesity**<br>Participants with a body mass index (BMI) of 25kg/m² or higher (obese) or between 23kg/m² and 25kg/m² (pre-obesity or overweight) at the 24-week follow-up evaluation of Phase 1 will transition to Phase 2. Participants will continue their psychotropic medication regimen and, based on the clinician's judgment, will receive one of the following anti-obesity medications for an additional 24 weeks: orlistat, naltrexone/bupropion combination, liraglutide, phentermine/topiramate combination, phentermine, semaglutide, metformin, or topiramate (phentermine will be administered for a maximum of 12 weeks). Anthropometric and body composition factors will be assessed 12 weeks after the initiation of anti-obesity medication, and all factors except demographic data will be reassessed after 24 weeks. For participants receiving phentermine, anthropometric and body composition factors will be assessed after 4 weeks, and the medication will be discontinued within 12 weeks, followed by a post-treatment evaluation.
Efficacy	An artificial intelligence algorithm will be developed to predict the likelihood of weight gain side effects from psychotropic medications and the effectiveness of anti-obesity treatments for each individual. This will be achieved by using deep learning to analyze and learn the changes caused by each psychotropic medication, anti-obesity medication, and their combinations on weight and obesity-related metabolic factors, including demographic factors, lifestyle factors, family history and comorbidities, anthropometric and body composition factors, hematologic factors, and psychological scales.
Safety	Occurrence of adverse events; names of adverse events; Severity; Occurrence and detailed information of serious adverse events; Causal relationship with the study drug, and rate of drug discontinuation due to adverse events.
Expectations and Expected results	An artificial intelligence algorithm will be developed to predict the occurrence of weight gain and metabolic side effects caused by psychotropic medications in individuals and to identify which anti-obesity treatments would be effective in cases of psychotropic medication-induced obesity.

Research protocols

1. Study title

Predicting the likelihood of weight gain as a side effect of psychiatric medications and the effectiveness of anti-obesity drugs: A pilot study

2. Name and address of the study site

Chung-Ang University Hospital, 102, Heukseok-ro, Dongjak-gu, Seoul, Korea (06973)

3. Principal investigator and co-investigator names and

1) **Principal Investigator:** Sun Mi Kim (Associate Professor)
2) **Sub-investigator:** Doug Hyun Han (Professor), Hye Jun Lee (Assistant Clinical Professor)
3) **Study Coordinator:** Na Yeon Kim (Fellow), Da Seul Kim (Postdoctoral Researcher)

4. **Study Sponsor**

1) **Name and address of study sponsor:** N/A
2) **Name and title of monitor(s**): N/A

5. Name and address of the funding organization

Chung-Ang University, 84, Heokseok-ro, Dongjak-gu, Seoul, Korea (06974)

6. Estimated study duration

From the IRB approval date for up to 36 months

7. Diseases under study

Depression, bipolar disorder, and schizophrenia

8. Background and purpose of the study

1) Research Background

Obesity is one of the most critical health issues, and the global obesity epidemic is adding to the burden of disease. In Asians, obesity is defined as a body mass index (BMI) of 25 kg/m2 or higher, and the prevalence of obesity among adults in Korea in 2022 was 37.2%. Obesity increases the co-morbidity of various chronic diseases, including metabolic syndrome such as type 2 diabetes, hypertension, and dyslipidemia, cardiovascular disease, cancer, gastrointestinal and gallbladder diseases, and musculoskeletal diseases, and the risk of social and mental disability and mortality. Therefore, to reduce the rapidly increasing morbidity and mortality associated with obesity, obesity management strategies must extend beyond the treatment of obesity to the proper prediction, management, and prevention of risk factors associated with weight gain.

Globally, the prevalence of obesity among individuals with severe mental illnesses such as schizophrenia, bipolar disorder, and major depressive disorder is 25.9%. When including those classified as overweight (pre-obesity: BMI 23–25kg/m^2), the prevalence rises to 60.1%, affecting more than half of this population. Compared to the general population, people with severe mental illness are 3.04 times more likely to be obese and 2.03 times more likely to be overweight or obese. The prevalence of metabolic syndrome in people with severe mental illness is also higher than in the general population at 26.1%. They have a mortality rate 2-3 times higher than the general population and a life expectancy that is 10-20 years shorter on average, with cardiovascular disease being the leading cause of death.

Certain antipsychotics, antidepressants, and mood stabilizers are widely known to commonly cause weight gain and metabolic changes as side effects. Among the top-prescribed antipsychotics in South Korea, olanzapine and quetiapine, in particular, have been shown to induce weight gain in a dose-dependent manner and affect

various metabolic markers. Aripiprazole, initially perceived to have fewer weight gain side effects due to its mechanism of action, has been increasingly reported to cause significant weight gain as clinical experience accumulates. Recent studies have confirmed this finding, drawing considerable attention and debate. A meta-analysis on antidepressant-induced weight gain revealed that, excluding tricyclic antidepressants (which are no longer commonly used as first-line treatments), mirtazapine poses the highest risk for weight gain. Furthermore, valproic acid, one of the most frequently prescribed mood stabilizers, has been reported to cause weight gain in up to 50% of patients. Weight gain caused by psychotropic medications is associated with an increased risk of metabolic syndrome, cardiovascular and cerebrovascular diseases, and higher mortality rates. It is also linked to reduced self-esteem, decreased quality of life, and increased healthcare costs. Additionally, weight gain is one of the primary reasons for poor treatment adherence in patients.

Although a variety of anti-obesity medications are widely prescribed, there is a lack of research on which medications are superior in terms of efficacy and side effects for individual patients. Non-personalized, uniform prescriptions may hinder treatment effectiveness, lead to misuse or overuse of anti-obesity medications, and result in unnecessary healthcare costs. The effectiveness and side effects of anti-obesity medications vary depending on numerous factors, including genetic predisposition, socioeconomic status, lifestyle, body composition, comorbidities, and concurrent medications. Therefore, research is needed to identify predictors of treatment effectiveness that comprehensively account for these factors.

Sensitivity to weight gain from psychotropic medications varies significantly among individuals, even when using the same medication. However, there is still a lack of research identifying predictors that can estimate the degree of weight gain in patients with psychiatric disorders undergoing psychotropic treatment. The risk of psychotropic medication-induced weight gain is complex and influenced by multiple factors, including genetic predisposition, lifestyle, body composition, and the severity

of psychiatric symptoms. Thus, comprehensive research on predictive factors for weight gain that considers these variables is essential. While recent studies have utilized machine learning to predict individual obesity risks in the general adult population, there have been no reported machine learning studies focusing on predicting the risk of weight gain from psychotropic medications in patients with psychiatric disorders.

2) Research hypothesis and objectives

This study aims to develop an artificial intelligence algorithm to predict the occurrence of weight gain and metabolic side effects caused by psychotropic medications in individuals and to identify the most effective anti-obesity treatments for cases of psychotropic medication-induced obesity.

9. drug and (in vitro diagnostic) medical device code name (or generic name of the main ingredient), amount of active pharmaceutical ingredient, formulation, etc.

(1) Psychotropic medications: Olanzapine, quetiapine, aripiprazole, mirtazapine, and valproate.

(2) Obesity medications: Orlistat, naltrexone/bupropion combination, liraglutide, phentermine/topiramate combination, phentermine, semaglutide, and metformin and topiramate prescribed for weight control.

10. Inclusion criteria, exclusion criteria, targeted number of participants and rationale for calculations

[Phase 1] Study on the potential for weight gain side effects of psychotropic drugs

1) Inclusion criteria

A. Adults aged 19 years or older

B. Individuals diagnosed with depression, bipolar disorder, or schizophrenia according to the DSM-5 diagnostic criteria for mental disorders

C. Individuals with no history of using olanzapine, quetiapine, aripiprazole, mirtazapine, or valproate within one month prior to the baseline evaluation

D. Individuals with no history of taking anti-obesity medications for weight loss within one month prior to the baseline evaluation

2) Exclusion criteria

A. Individuals with current severe medical conditions (e.g., cancer, heart failure, renal failure, liver disease, lung disease, thyroid disease, acute inflammatory conditions, etc.)

B. Individuals with a past or current history of substance dependence or misuse

C. Individuals with a history of epilepsy, head trauma, or organic mental disorders

[Phase 2] Study on predicting the effectiveness of anti-obesity drugs in psychotropic drug-induced obesity

1) Inclusion Criteria

A. Individuals classified as obese (BMI ≥ 25kg/m²) or pre-obese/overweight (BMI between 23kg/m² and 25kg/m²) at the conclusion of Phase 1.

2) Exclusion Criteria

Same as Phase 1

3) The number of people you want to reach

300 participants

4) Rationale for sample size calculation

A previous study using machine learning and deep learning techniques to

develop an individualized pharmacotherapy model for valproic acid in bipolar disorder patients (Zheng, et al., 2022) reported an accuracy of 73%–85% using a final dataset of 164 cases extracted from electronic medical records. Similarly, a study predicting weight gain risks from antipsychotics, antidepressants, and mood stabilizers using machine learning (Eder, et al., 2024) included 163 patients and reported an average accuracy of 66.05%–79.62%. However, the dropout rate after four weeks was high at 37%, leaving 103 cases with confirmed weight changes. To secure a dataset comparable to these studies, this study plans to recruit 300 participants, considering an estimated dropout rate of approximately 40%.

5) Plan for recruiting study participants

This study will recruit participants by posting a recruitment notice in the Department of Psychiatry at Chung-Ang University Hospital and distributing study information to outpatients and inpatients. The study will be explained to individuals interested in participating, and those who voluntarily consent will be screened to ensure they meet the inclusion and exclusion criteria before proceeding with the study.

6) Assessment of Research Participants' Capacity to Consent

To evaluate whether participants fully understand the study and can voluntarily consent, all participants will be assessed to ensure they comprehend the nature, purpose, procedures, potential risks, and benefits of the study before providing consent.

(1) Assessment Procedure:

Researchers will explain the study in an easy-to-understand manner and verify participants' understanding through open-ended questions about the study's purpose, procedures, potential risks and benefits, and their right to withdraw

at any time.

(2) Assessment Criteria:

Participants will be deemed capable of providing informed consent if they meet the following criteria:

- Understanding the information about the study
- Ability to clearly communicate their decision
- Making consistent decisions aligned with their own values and preferences

11. Research methods

1) Specific research methods

- **[Phase 1]** Demographic factors, lifestyle factors, family history and co-morbidities, anthropometric and body composition factors, hematologic factors, and psychometric scales will be measured at baseline in recruited participants.
- If participants are already taking psychotropic medications, they will continue their current regimen while being additionally prescribed one of the following medications known to have significant weight gain side effects—olanzapine, quetiapine, aripiprazole, mirtazapine, or valproic acid—for 24 weeks.
- Anthropometric and body composition factors will be assessed 12 weeks after the initiation of psychotropic medication, and all factors except demographic factors will be reassessed at 24 weeks.

- **[Phase 2]** At the 24-week follow-up evaluation in Phase 1, participants with a BMI of 25kg/m² or higher (obese) or between 23kg/m² and 25kg/m² (pre-obesity/overweight) will transition to Phase 2. (Participants meeting the eligibility criteria for Phase 2 will be asked to confirm their willingness to continue in the study before proceeding.)
- Participants will maintain their psychotropic medication regimen and, based

on the clinician's judgment, will be prescribed one of the following anti-obesity medications for an additional 24 weeks: orlistat, naltrexone/bupropion combination, liraglutide, phentermine/topiramate combination, phentermine, semaglutide, metformin, or topiramate (phentermine will be administered for a maximum of 12 weeks).

- Anthropometric and body composition factors will be assessed 12 weeks after initiating anti-obesity treatment, and all factors except demographic data will be reassessed at 24 weeks. For participants prescribed phentermine, anthropometric and body composition factors will be assessed at 4 weeks, with the medication discontinued within 12 weeks, followed by a post-treatment evaluation.

Study Flow

Steps	Phase 1				Phase 2	
**Item**	**Screening**	**Baseline**	**F/U**	**F/U**	**F/U**	**F/U**
**Visit**	**V0**	**V1**	**V2**	**V3**	**V4**	**V5**
	**-2wk~**	**D0**	**12wk±7d**	**24wk 7d±**	**36wk 7d±**	**48wk 7d±**
Written consent	●					
Inclusion/exclusion criteria	●					
Demographic Information	●	●				
Medication prescription		●	●	●	●	●
Adherence assessment			●	●	●	●
Dietary survey		●		●		●
Korean Eating Attitudes Test-26 (KEAT-26)		●		●		●

Short-form International Physical Activity Questionnaire (IPAQ)		●		●		●
Alcohol Use Disorders Identification Test (AUDIT-K)		●		●		●
Insomnia Severity Inventory (ISI-K)		●		●		●
Family history and comorbidities assessment		●		●		●
Anthropometric and body composition analysis		●	●	●	●	●
Blood tests		●		●		●
Positive and Negative Syndrome Scale (PANSS)		●		●		●
Beck Depression Inventory (BDI-II)		●		●		●
Mood Disorder Questionnaire (MDQ)		●		●		●
* Visit 1 and Visit 2 can take place on the same day						

2) Setting up a comparison group and randomization method

N/A

3) Dosage and Administration of Investigational Drugs, Methods of Use,

Combination Therapies, and Rationale for Selecting Control Drugs

(1) Study Drug

- [Phase 1] Psychotropic medications: Olanzapine, quetiapine, aripiprazole, mirtazapine, valproic acid
- [Phase 2] Anti-Obesity Medications: Orlistat, naltrexone/bupropion combination, liraglutide, phentermine/topiramate combination, phentermine, semaglutide, metformin (prescribed for weight control), or topiramate

(2) Standard Guidelines for Anti-Obesity Medications

- The 8th Edition of the Korean Society for the Study of Obesity's Clinical Practice Guidelines for Obesity (2022) recommends the following principles for pharmacotherapy: (1) The foundational treatments for obesity include dietary therapy, exercise therapy, and behavioral therapy. Pharmacotherapy should be used as an adjunctive treatment alongside these approaches. (2) Pharmacotherapy should be considered for patients with a BMI of 25kg/m² or higher who have failed to achieve weight loss through non-pharmacological methods. (3) For long-term weight management, medications approved based on results from large-scale clinical trials should be used. (4) If a weight loss of at least 5% is not achieved within three months of maintaining the therapeutic dose of anti-obesity medication, it is recommended to change or discontinue the medication.
- Regarding the indications for pharmacotherapy in obesity, the U.S. National Institutes of Health (NIH) recommends initiating pharmacological treatment for patients with a BMI of 30kg/m² or higher, or for those with a BMI of 27kg/m² or higher if they have high cardiovascular risk factors such as hypertension, diabetes, dyslipidemia, or sleep apnea. The Asia-Pacific Guidelines for Obesity Treatment suggest considering pharmacotherapy for patients with a BMI of 25kg/m² or higher, or for those with a BMI of 23kg/m²

or higher if they are at high risk for cardiovascular disease. In South Korea, pharmacotherapy is recommended for patients with a BMI of 25kg/m² or higher who have failed to lose weight through non-pharmacological methods. However, these recommendations differ from the official drug approvals currently aligned with NIH standards. Therefore, if a clinician intends to prescribe anti-obesity medications outside of the officially approved indications, they must discuss the situation thoroughly with the patient, explain both the benefits and potential risks of the treatment, and obtain informed consent before proceeding.

(3) Dosage and Administration

- The type and dosage of psychotropic medications and anti-obesity medications prescribed to each participant will be determined by a specialist based on medication guidelines and medical judgment. Dosages will be adjusted within the maximum allowable dose.

※ Prescription of anti-obesity medications

- In South Korea, anti-obesity medications are only prescribed on a non-reimbursable basis, with monthly costs generally ranging from 200,000 to 400,000 KRW depending on the medication and dosage.
- **Phentermine:** As a short-term treatment for obesity, the recommended duration of use is **within four weeks**. If satisfactory weight loss is achieved within this period, treatment may be continued, but the total prescription duration **should not exceed three months**. The usual recommended dose is 15–37.5mg, taken before breakfast or 1–2 hours after breakfast.
- Long-term Anti-Obesity Medications: The treatment period varies depending on the individual but is generally conducted for 6–10 months.
- **Orlistat:** Approved for long-term weight management, the recommended **dosage is 120mg three times daily before meals**. Orlistat prevents the

absorption of approximately 30% of dietary fat when taken at the recommended dose, with no additional benefit from higher doses.

- **Naltrexone/Bupropion Combination:** Approved for long-term use, the dosage is gradually increased over four weeks: **one 8/90mg tablet in the morning during the first week, one tablet in the morning and one in the evening during the second week, two tablets in the morning and one in the evening during the third week, and two tablets in the morning and two in the evening from the fourth week onward**. The maximum daily dosage is 32mg of naltrexone and 360mg of bupropion, maintained at a dose appropriate for the patient.
- **Phentermine/Topiramate Combination:** Approved for long-term weight management, commonly used maintenance dosages are 7.5/46mg or 15/92mg once daily. Treatment typically starts with a low dose of **3.75/23mg once daily for the first 14 days** to improve tolerability, followed by an **increase to 7.5/46mg for 12 weeks**. If a 3% reduction in baseline weight is not achieved after 12 weeks, the dose may be **increased to 11.25/69mg for 14 days, then to 15/92mg**. The medication is taken once daily in the morning, with or without food, to avoid insomnia.
- **Liraglutide:** Administered as a **subcutaneous injection once daily, starting at 0.6mg for the first week and gradually increasing by 0.6mg at weekly intervals.** The maximum dose of 3.0mg is maintained thereafter.
- **Metformin:** Although the dosage range for weight loss has not been established, typical dosages range from 500mg to 2,000mg daily. The maximum dose for diabetes treatment is 850mg three times daily. Patients typically begin with **500mg two to three times daily**, with a **gradual weekly increase of 500mg** to assess the drug's effectiveness.
- **Topiramate:** Starts at a low dose and is taken once daily, regardless of meals. Weight loss effects are significant at doses of 100–200mg/day when

taken for more than one month, with weight loss also observed at the initial dose of 25mg/day. Typically, 25mg is taken in the evening during the first week, followed by **increases of 25–50mg every 1–2 weeks**. The recommended daily dose is 100–200mg, with a maximum daily dose of 500mg.

- **Semaglutide:** Administered as a **subcutaneous injection once weekly, starting at 0.25mg per week**. The dosage is gradually increased approximately every four weeks, reaching a maintenance dose of 2.4mg per week.

※ Selection of Anti-Obesity Medications Based on Comorbidities

Table 1. Selection of Anti-Obesity Drugs Based on Comorbidities

Comorbidities	Orlistat	Naltrexone / bupropion ER	Liraglutide 3.0 mg	Phentermine / topiramate ER
Type 2 Diabetes				
Hypertension		Blood pressure and pulse monitoring are recommended<br>Contraindicated in uncontrolled hypertension	Pulse monitoring is recommended	Pulse monitoring is recommended
Cardiovascular Disease		Blood pressure and pulse monitoring are recommended		
Chronic Kidney Disease (Mild: 60–89 mL/min)				
Chronic Kidney Disease (Moderate: 30–59 mL/min)		Should not exceed 8 mg/90 mg bid/day		Should not exceed 7.5 mg/46 mg/day

Chronic Kidney Disease (Severe: <30 mL/min)	Caution: Risk of oxalate	Should not exceed 8 mg/90 mg bid/day		Should not exceed 7.5 mg/46 mg/day
		Not recommended in end-stage renal disease		Not recommended in end-stage renal disease
Liver Dysfunction (Mild to moderate: Child-Pugh 5–9)	Caution: Risk of gallstones	Should not exceed 8 mg/90 mg/day	Caution: Risk of gallstones	Should not exceed 7.5 mg/46 mg/day
Liver Dysfunction (Severe: Child-Pugh >9)				
Obstructive Sleep Apnea				15 mg/92 mg/day recommended
Depression				Should not exceed 7.5 mg/46 mg/day
Glaucoma				
Pancreatitis				

■Green: Recommended for Use

■Yellow: Use with Caution

■Orange: Not Recommended

■Gray: Insufficient Evidence

Reference: Korean Society for Obesity, Obesity Treatment Guidelines Summary 8th Edition (2022).

4) Observation Items, Clinical Examination Items, and Observation Methods

㉠ Demographic factors: gender, age, education, income level, marital status, occupation, etc.

㉡ Lifestyle factors: diet (nutrition), exercise (physical activity), alcohol consumption, and sleep, etc.

- Diet: Participants will record their meals for 3 days over the past week on days resembling their usual eating patterns. Total energy and nutrient intake

will be calculated. Eating-related issues will be assessed using the Korean Version of the Eating Attitudes Test-26 (KEAT-26, 26 items).

- Exercise: Physical activity will be evaluated using the Korean version of the Short International Physical Activity Questionnaire (IPAQ, 7 items).

- Alcohol Consumption: Alcohol use will be assessed using the Korean version of the Alcohol Use Disorder Identification Test (AUDIT-K, 10 items).

- Sleep: Sleep will be evaluated using the Korean version of the Insomnia Severity Index (ISI-K, 5 items).

ⓒ Family History and Comorbidity Factors: Family history of obesity, comorbidities (including metabolic syndrome), and history of obesity medication use.

ⓡ Anthropometric and Body Composition Factors: Height, waist circumference, waist-to-hip ratio will be measured. Body composition, including weight, body mass index (BMI), body fat mass, lean body mass, muscle mass, and abdominal fat percentage, will be measured using bioelectrical impedance analysis (BIA).

ⓜ Hematological Factors: Levels of insulin, fasting blood glucose, lipids (total cholesterol, low-density lipoprotein cholesterol, high-density lipoprotein cholesterol, triglycerides), uric acid, and high-sensitivity C-reactive protein will be measured.

ⓑ Psychometric Scale Factors: Schizophrenia symptom Score, Depression Score, Bipolar disorder scale Score

- Positive and Negative Syndrome Scale (PANSS, 30-item): Structured interviews will be used to assess positive and negative symptoms of schizophrenia.

- Beck Depression Inventory-II (BDI-II, 21-item): The most widely used scale for evaluating depressive symptoms.

- Mood Disorder Questionnaire (MDQ, 15-item): A screening tool for bipolar spectrum disorders, used in clinical practice to assess symptom severity.

ⓢ Electrocardiogram, pregnancy test: Conducted during [Phase 2] prior to

prescribing anti-obesity medications, only when necessary to confirm contraindications.

5) Efficacy evaluation criteria and methods

The primary efficacy criterion is the development of an artificial intelligence (AI) algorithm capable of predicting:

- The occurrence of weight gain and metabolic side effects induced by psychotropic medications in individuals.
- The effectiveness of specific anti-obesity medications in addressing obesity caused by psychotropic medications.

6) Differences from Existing Treatments and Research

Recent studies utilizing machine learning have focused on predicting individual obesity risk in the general adult population. However, no machine learning studies have yet been reported on predicting the risk of weight gain induced by psychotropic medications in individuals with mental disorders. By incorporating such predictions into medical decision-making, it would be possible to select the most optimal psychotropic medication from the outset of treatment planning. This approach is expected to not only improve mental health symptoms but also prevent obesity and metabolic syndrome, thereby effectively managing physical health.

Additionally, selecting the most appropriate anti-obesity medication for patients with mental disorders diagnosed with obesity is expected to facilitate rapid treatment of obesity and metabolic syndrome, prevent complications, and enhance long-term adherence to psychotropic medication treatment. Consequently, this would also be advantageous for managing mental health symptoms in the long term.

7) Benefits and risks for research participants

(1) Benefits: There are no direct benefits to participants from participating in

this study. However, participants will be able to track changes in their health status through test and evaluation results at each measurement point. Additionally, they will receive feedback on the results of the items assessed in this study. The findings of this study will contribute to the development of an algorithm that predicts the likelihood of weight gain as a side effect of psychotropic medications and the effectiveness of anti-obesity treatments.

(2) Risks: The psychotropic medications used in this study are generally known to cause weight gain as a side effect. Among anti-obesity medications, appetite suppressants approved for short-term use, such as **phentermine**, may activate the sympathetic nervous system, potentially causing side effects such as dizziness, insomnia, anxiety, dry mouth, increased blood pressure, tachycardia, palpitations, primary pulmonary hypertension, or valvular heart disease. **Orlistat** may frequently cause gastrointestinal side effects such as fecal incontinence, fatty stools, and abdominal bloating, and in rare cases, liver damage has been reported. Common side effects of **liraglutide** include gastrointestinal symptoms (vomiting, diarrhea, constipation, indigestion, abdominal pain) and gallbladder-related symptoms (gallstones, cholecystitis). It is contraindicated in individuals with a history of pancreatitis, medullary thyroid cancer, or multiple endocrine neoplasia syndrome. The most common side effects of **naltrexone/bupropion** include nausea, vomiting, constipation, headache, dizziness, sleep disturbances, and dry mouth. Patients with mood disorders and mental illnesses taking antipsychotics or antidepressants require caution due to the risk of drug interactions and seizures. In patients taking bupropion with norepinephrine reuptake inhibitors (SNRIs), serotonin syndrome, delirium, agitation, insomnia, increased heart rate and blood pressure, hypomania, and suicidal ideation have been reported. Similarly, the combination of bupropion with selective serotonin reuptake inhibitors (SSRIs) has been associated with serotonin syndrome, seizures, delirium, insomnia,

dizziness, and gastrointestinal adverse effects. It is contraindicated for individuals with bipolar disorder, abrupt withdrawal from alcohol, benzodiazepines, barbiturates, or antiepileptic drugs, as well as those with bulimia or anorexia nervosa. **Phentermine/topiramate** is commonly associated with paresthesia, dizziness, dry mouth, constipation, taste alteration, insomnia, and anxiety. Abrupt discontinuation may induce seizure symptoms; therefore, it must be tapered gradually. **Topiramate** has been reported to cause neuropsychiatric adverse effects such as impaired concentration/attention, dizziness, agitation, emotional instability, and depression. Anticonvulsants, including topiramate, may increase suicidal ideation, necessitating monitoring for new or worsening depression, suicidal thoughts, or changes in mood or behavior. Topiramate may also enhance the toxicity of lithium, valproic acid, and valproate semisodium, which are classified as grade 1 severe adverse effects, and concurrent use should be avoided. **Metformin** may cause lactic acidosis (symptoms: fatigue, muscle pain, abdominal discomfort, shortness of breath) and hypoglycemia, and common side effects include nausea, vomiting, abdominal bloating, loss of appetite, indigestion, constipation, and abdominal pain. Additional adverse effects include skin rash and vitamin B12 deficiency. **Semaglutide** has been frequently reported to cause complications such as diabetic retinopathy, headaches, vomiting, abdominal pain, diarrhea, and hypoglycemia. Rarely, it may lead to acute pancreatitis and hypotension. No interactions between semaglutide and psychiatric medications have been reported, and it is considered safe for concurrent use. However, semaglutide may delay gastric emptying and affect the absorption of oral medications. Other inconveniences may include time commitments for various tests, and blood sample collection may cause pain, bruising, or dizziness. If participants experience such discomfort, the principal investigator or co-researchers will conduct a

thorough examination to seek appropriate solutions. The research team will always strive to minimize risks and discomfort for participants and ensure that medical treatment is provided when necessary.

※ Adverse Reactions, Precautions, and Contraindications for Anti-Obesity Medications

Duration	Classification	Medications (example)	Adverse Reactions	Precautions	Contraindications
Short-Term	Sympathomimetic Appetite Suppressant	Phentermine (Phurimin Tablet)	Dry mouth, paresthesia, constipation, dizziness, blurred vision, eye pain, insomnia.	- Not recommended for patients who have used other appetite suppressants within the past year.<br>Abrupt<br>- Discontinuation may cause extreme fatigue, depression, sleep disturbances, or ECG changes.<br>- Adjust dosage when used with oral diabetes medications.<br>- Late-night administration may induce insomnia.	- Not for use in individuals under 16 years of age.<br>Contraindicated in pregnant women (DUR grade 1) and breastfeeding women.<br>- Concomitant use with other appetite suppressants is prohibited.<br>- Contraindicated in patients with cardiovascular or cerebrovascular diseases.<br>- Not recommended for patients with moderate to severe hypertension (including pulmonary hypertension).<br>- Contraindicated in patients with moderate to severe kidney or liver dysfunction.<br>- Not suitable for

					those with severe heart or pancreatic disease.<br>- Contraindicated in patients with glaucoma.<br>- Not for use in individuals with a history of drug or alcohol abuse.<br>- Contraindicated in patients taking MAOIs within the past 14 days.<br>- Not suitable for patients with arterial occlusion.<br>- Contraindicated in those with hyperthyroidism.<br>- Not recommended for patients experiencing severe anxiety or agitation.
Long-term Therapeutics	Lipase Inhibitors	Orlistat (Xenical Capsules) Ages 12 and up (FDA)	- Abdominal bloating, flatulence, fatty stools.	- May reduce the absorption of fat-soluble vitamins. It is recommended to take multivitamins at a 2-hour interval from Orlistat.<br>- If taken with cyclosporine, administer with a 3-hour interval to avoid interactions.	- DUR grade 1 contraindicated in pregnancy.<br>- Chronic malnutrition.<br>- Gallbladder disease.
	GLP-1 Receptor Agonist	Liraglutide (Saxenda Injection) 12 years of age or older	- Nausea, vomiting, diarrhea, constipation, abdominal pain.<br>- Headache,	- Increased risk of suicidal ideation; monitor for changes in mood or behavior.<br>- Risk of acute	- DUR grade 2 contraindicated in pregnancy.<br>- Personal or family history of medullary thyroid

			dizziness, fatigue.<br>- Hypoglycemia, dehydration.<br>- Cholecystitis, pancreatitis.	biliary diseases and increased blood pressure.<br>- Not to be used as a substitute for insulin.<br>- When used with sulfonylureas, monitor for increased risk of hypoglycemia.<br>- Frequent INR monitoring required when co-administered with warfarin.	carcinoma (MTC) or multiple endocrine neoplasia syndrome type 2 (MEN2).
	Compounding Agents	Phentermine/Topiramate (Qsymia Capsule)<br>18 years of age or older	- Altered taste/sensation, paresthesia, dizziness, anxiety, dry mouth, insomnia.	- Teratogenicity: Prescribe only after confirming a negative pregnancy test.<br>- Gradual tapering is necessary upon discontinuation to reduce the risk of seizures.<br>- Risk of hypoglycemia; listed as a doping substance.<br>- Contraindicated with certain medications (phendimetrazine, mazindol, selegiline, bupropion, naltrexone, diethylpropion).	- DUR grade 1 contraindicated in pregnancy; not for use in breastfeeding women.<br>- Coronary artery disease.<br>- Uncontrolled hypertension. Glaucoma.<br>- Concurrent use of MAOIs within 14 days.<br>- Hyperthyroidism.<br>- Severe agitation or anxiety.
		Naltrexone/Bupropion (Contrave Extended-	- Nausea, vomiting, constipation, headache,	- Risk of suicidal ideation; monitor blood pressure and heart rate.	- DUR grade 1 contraindicated in pregnancy; not for use in

		Release Tablet) For adults under 75 years of age	dizziness.<br>- Insomnia, dry mouth.<br>- Increased blood pressure, tachycardia, elevated intraocular pressure, visual field defects.	- Increased risk of seizures.<br>- May induce closed-angle glaucoma.<br>- Potential hepatotoxicity.<br>- Prevention of kidney stones requires sufficient water intake.	breastfeeding women.<br>- Epilepsy, liver dysfunction, end-stage renal disease.<br>- Bipolar disorder, opioid dependency.<br>- Uncontrolled hypertension.<br>- Use of MAOIs within the last 14 days.<br>- Abrupt discontinuation of alcohol, benzodiazepines, barbiturates, or antiepileptic drugs.<br>- Patients with bulimia or anorexia nervosa.

Sources: State of the art in the treatment of obesity - focusing on the American Society for Obesity 2022 practice guidelines (2023), Pharmacy Information Center PharmReview

8) Discontinuation and Dropout Criteria

The participation of research subjects will be immediately discontinued under the following circumstances:

① If the research subject expresses a refusal to continue participation.

② If the subject discontinues prescribed medication for more than 2 weeks without consulting the medical staff during the study.

③ If clinical findings arise during the trial that warrant exclusion of the subject.

④ If other unavoidable circumstances prevent further participation in the clinical trial.

9) Safety Evaluation Criteria, Methods, and Reporting Procedures Including Adverse Events

▶ Definitions of Safety-Related Terms

① Adverse Event (AE): An undesirable and unintended sign, symptom, or disease that occurs after the administration of the investigational drug. AEs do not necessarily have a causal relationship with the investigational drug.

② Serious Adverse Event (SAE): An AE occurring at any dose of the investigational drug that meets one or more of the following criteria:

- Death of the research participant.
- Life-threatening conditions (events where the participant was in immediate danger of death at the time of occurrence).
- Persistent or significant disability or functional impairment.
- Requires hospitalization or prolongation of existing hospitalization.
- Causes congenital anomalies or birth defects.
- Other medically significant conditions.

Even if not explicitly listed above, situations judged by medical experts to have a significant impact on the safety and well-being of the research participant may be considered an SAE. Appropriate actions will be taken based on expert medical judgment.

- Product Quality Complaint (PQC):

Information provided by healthcare professionals, consumers, sales representatives, regulatory authorities, partner/collaborating companies, local branches, or other third parties regarding defects in the identification, quality, durability, reliability, safety, efficacy, or performance of a drug or suspected counterfeit drug. PQCs may include safety-related information but are not limited to it.

▶ Evaluation Methods

- All safety-related information occurring from the initiation of the investigational drug administration until 28 days after the end of administration must be thoroughly recorded in the Adverse Event Report

Form. Any unrecorded information will be classified as subjective symptoms.

- The severity of adverse events will be evaluated by the research staff based on predefined evaluation criteria. It is mandatory to assess and categorize symptoms according to their severity level in a stepwise manner.

▶ Evaluation Criteria

* Severity of Adverse Events
  - Mild: Adverse events that are easily tolerable
  - Moderate: Adverse events that significantly interfere with daily activities
  - Severe: Adverse events that prevent normal daily activities

* Outcome
  - Complete recovery (no sequelae)
  - Recovery with sequelae
  - Ongoing
  - Permanent damage
  - Death

* Actions taken for investigational drugs
  - Drug interrupted
  - Drug withdrawn
  - Dose reduced
  - Dose increased
  - Not applicable

▶ Causality Assessment with Investigational Drug

In the event of an adverse reaction, the principal investigator will classify the relationship between the investigational drug and the adverse reaction as follows. If necessary, the opinion of the research staff will be added:

  - Definitely related
  - Probably related
  - Possibly related

- Probably not related
- Definitely not related
- Unknown

▶ Reporting Procedures

① Reporting Adverse Events

- Education and Reporting by Participants: The principal investigator provides education to research staff, participants, or their guardians regarding all potential adverse reactions that may occur after drug administration. Participants are instructed to report any and all observed phenomena post-administration.
- Documentation: All systemic or clinical pathological symptoms observed after drug administration, including type, onset time, severity, treatment, therapeutic drugs, progress, and causality with the investigational drug, must be recorded in the Case Report Form (CRF).
- Evaluation and Reporting: The principal investigator documents and evaluates all symptoms occurring during the study period in the final study report. If a Serious Adverse Event (SAE) occurs, it must be reported to the Institutional Review Board (IRB) to determine whether the study should continue or be terminated.
- Follow-Up Reporting: Periodic safety information reports must be submitted until the adverse event is resolved (e.g., resolution of symptoms or inability to continue follow-up).
- Compliance with Ethical Guidelines:
- All aspects of the clinical trial are conducted in compliance with the Declaration of Helsinki.

② Expedited Reporting

- Purpose: To promptly inform investigators and related parties about new and critical information regarding serious adverse events.

- Subjects of Reporting: Serious adverse events, unexpected adverse events, and information that could generally impact the risk-benefit assessment of the investigational drug (e.g., significant safety findings from newly conducted animal studies) or information that may result in changes to the administration of the investigational or comparator drug fall under this category.

- Reporting Timeline: In the event of such an occurrence, the principal investigator must report it within the reporting period stipulated by the Institutional Review Board (IRB) to determine whether the study will continue or be discontinued.

- Reporting Method: For expedited reporting of serious adverse events, the designated serious adverse event reporting form provided by the Institutional Review Board (IRB) must be used. Even if it is not possible to obtain all the information at the time of expedited reporting, every effort must be made to gather as much information as possible.

▶ Actions to Take in the Event of Serious Adverse Events or Quality Complaints

During the study period, the principal investigator and research personnel must prioritize the safety of study participants. In the event of serious adverse events or quality complaints, prompt and appropriate measures must be taken to minimize the occurrence of serious adverse events.

The responsibilities of each party during the clinical trial are as follows:

① Responsibilities of the Principal Investigator

The investigator must report to the IRB as soon as they become aware of the event, following the reporting timeline outlined in institutional regulations. If necessary, the clinical trial involving the investigational product may need to be partially or entirely suspended.

② Responsibilities of the Research Personnel

The investigator must report the event to the principal investigator and sponsor within 24 hours of becoming aware of it.

③ Responsibilities of the IRB

Upon receiving a report of a serious adverse event, the IRB must take necessary actions, such as issuing an order to partially or entirely suspend the clinical trial, and communicate this decision to the principal investigator.

④ Responsibilities of the Sponsor

In the case of multi-center clinical trials, the sponsor must immediately notify other participating institutions of any serious adverse events reported by the affected institution.

* Suicide Risk Monitoring

Investigators must consider monitoring for suicide risk throughout the trial.

10) Data Safety Monitoring Plan (DSMP)

- This study involves the use of an already marketed drug at an approved dosage with a low frequency of adverse events. Therefore, it corresponds to Level II (slightly above minimal risk).
- Monitoring Lead: Associate Professor Sun Mi Kim, Department of Psychiatry.
- Monitoring Frequency: During the participant recruitment and follow-up period, monitoring will occur at least once every six months.
- Reporting in the Absence of Serious Adverse Events: If no serious adverse events occur within the institution, the monitoring results will be included in the final report (Safety Information Report).
- Reporting in the Event of Serious Adverse Events: In the case of serious adverse events, the details will be reported according to the standard procedures of the Participant Protection Center using the "Adverse Drug/Medical Device Reaction Report Form (Domestic/International Use)".
- If a single case of a serious adverse event involving death or life-threatening

conditions is reported, or if three or more cases of other serious adverse events with probable or definite causality are identified, the principal investigator must consult with co-investigators regarding early termination of the study and report the outcome to the Participant Protection Center.

11) Data analysis and statistical methods

This study aims to develop an artificial intelligence algorithm to predict the likelihood of weight gain side effects associated with individual psychotropic medications and the effectiveness of anti-obesity treatments. The changes in weight and obesity/metabolism-related factors caused by various combinations of psychotropic medications and anti-obesity treatments will be trained through deep learning, enabling the development of a predictive AI algorithm.

▷ Deep learning model development process

- Chung-Ang University's College of Engineering AI Graduate School has been designated to develop the deep learning model and AI algorithm.

㉠ Data augmentation

- Application of data augmentation techniques for small datasets:
  · Use of contrastive learning methods like SimSiam, combined with weighted augmentation for diverse views.
  · Expansion of tabular data using Variational Autoencoder for synthetic data generation.

㉡ Deep learning-based model design

- Tree-Based Approach:
  · Facilitates easy interpretation of decision-making processes and inference results.
  · Enables fast training and inference speeds.
- Transformer-Based Approach:

· Suitable for learning complex patterns in non-linear relationships and high-dimensional data.

· Capable of integrating and processing various types of data, such as images, text, and speech.

- Hybrid Model Design Combining Advantages of Both Methods:

· Developing specialized models for tabular data by integrating tree-based and transformer-based methods (e.g., TabNet, TabLLM).

ⓒ Deep learning model training and evaluation

- Applying the Attention mechanism to consider the temporal characteristics of each factor.

- Using Model-Agnostic Meta-Learning (MAML) to enhance model generalization performance and data efficiency.

- Evaluating model performance based on metrics such as RMSE to assess variations in key factors.

12) Study timeline

- IRB Approval to Month 30: Participant recruitment and collection of research data from recruited participants.

- Month 31 to Month 36: Data preprocessing, development of the deep learning model, and AI algorithm.

12. Measures to ensure the safety and protection of research participants

1) Basic measures to ensure ethical conduct of research

- All participating researchers have completed clinical ethics training within the past two years. The study will be conducted in accordance with the content of this training. The principal investigator will comply with domestic laws, including the Bioethics and Safety Act, as well as international guidelines such as the Declaration of Helsinki. Efforts will be made to respect the dignity,

rights, safety, and well-being of research participants and to ensure that the study's outcomes benefit humanity worldwide.

- A detailed explanation of the study will be provided to participants, and a copy of the consent form containing study information will be given to them.
- If any abnormal findings are discovered during examinations, participants will be informed without delay and efforts will be made to ensure they receive appropriate information and treatment.

2) Consent process for research participants

· Researchers Providing Explanation and Obtaining Consent: Associate Professor Sun Mi Kim (Department of Psychiatry), Professor Doug Hyun Han (Department of Psychiatry), Assistant Clinical Professor Hye Jun Lee (Department of Family Medicine), Fellow Na Yeon Kim (Department of Psychiatry).

· Individual Providing Consent: Research participant.

· Waiting Period Between Explanation and Consent Acquisition: If a research participant expresses their intention to consent more than 12 hours after the initial explanation, the study will be re-explained to the participant and their representative.

· Methods to Minimize Coercion or Undue Influence: Obtaining the consent of the participant or their representative is mandatory for all aspects of study participation. Even after recruitment, the designated research personnel will explain the study details again to ensure participants fully understand their involvement and prevent any research activities from proceeding without their clear comprehension.

· Language Used by Researchers During Explanation and Consent Process: Korean.

· Language Understandable to Research Participants or Their Representatives: Korean.

· Information and Consent Form Provided to Research Participants or Their Representatives: Refer to the study information sheet and consent form.

3) Compensation Plan for Research Participants

Participants will receive 20,000 KRW for transportation expenses upon completing the baseline assessment in Phase 1 and an additional 20,000 KRW upon completing the follow-up assessment at 24 weeks. For those proceeding to Phase 2, an additional 20,000 KRW will be provided upon completing the follow-up assessment at 24 weeks (48 weeks from the baseline assessment). Therefore, participants completing only Phase 1 will receive a total of 40,000 KRW, while those completing both Phase 1 and Phase 2 will receive a total of 60,000 KRW for transportation expenses. The costs of the assessments will be covered by the researchers through a research grant provided by Chung-Ang University.

4) Measures for Protecting the Privacy of Research Participants

During participation in this study, data collected from participants will include demographic information (e.g., gender, age, education level, income level, marital status, occupation), lifestyle habits (e.g., diet, exercise, alcohol consumption, smoking), family history and comorbidities, physical measurements and body composition data, blood test results, and responses to clinical scale items. Additionally, participants' phone numbers and bank account information will be collected during recruitment and compensation payment processes, but this information will be immediately discarded after compensation is provided.

Collected medical records, test results, health information, and research findings derived from data analysis may be reviewed by regulatory government agencies, funding organizations, or the hospital's Institutional Review Board in accordance with applicable laws to verify clinical trial

procedures and data quality. They may also be cited in other related research or publications. However, participants' identifiable information will remain confidential, and health data will be provided in a de-identified format.

If a participant withdraws consent for study participation, data collected up to the point of withdrawal may be used within the limits permitted by law. In accordance with Article 15 of the Enforcement Rule of the Bioethics and Safety Act, study-related records will be securely stored for three years in the Psychiatry Research Laboratory of Chung-Ang University Hospital after the study's completion. The storage cabinet will be equipped with a lock for security, and access will be limited to a small number of authorized personnel. Any documents containing personal or sensitive information will be destroyed in accordance with Article 16 of the Enforcement Decree of the Personal Information Protection Act once the retention period has expired.

5) Additional Protective Measures for Vulnerable Research Participants

N/A

13. Methods for Storage and Disposal of Human-Derived Materials

N/A

14. References

Afzal, M., Siddiqi, N., Ahmad, B., Afsheen, N., Aslam, F., Ali, A., ... Khalid, H. (2021). Prevalence of overweight and obesity in people with severe mental illness: systematic review and meta-analysis. Frontiers in Endocrinology, 12, 769309.

Beck, A. T., Steer, R. A., & Brown, G. (1996). The Beck depression inventory-II. San Antonio, 78(2), 490-498

Boland, R., Verdiun, M. and Ruiz, P. (2022). Kaplan & Sadock's synopsis of psychiatry, 12th edition. Wolters Kluwer.

Carrà, G., Bartoli, F., Carretta, D., Crocamo, C., Bozzetti, A., Clerici, M., & Bebbington, P. E. (2014). The prevalence of metabolic syndrome in people with severe mental illness: a mediation analysis. Social Psychiatry and Psychiatric Epidemiology, 49, 1739-1746.

Eder, J., Glocker, C., Barton, B., Sarisik, E., Popovic, D., Lämmermann, J., ... & Musil, R. (2024). Who is at risk for weight gain after weight-gain associated treatment with antipsychotics, antidepressants, and mood stabilizers: A machine learning approach. *Acta Psychiatrica Scandinavica*.

Ferdowsy, F., Rahi, K. S. A., Jabiullah, M. I., & Habib, M. T. (2021). A machine learning approach for obesity risk prediction. Current Research in Behavioral Sciences, 2, 100053.

Gill, H., Gill, B., El-Halabi, S., Chen-Li, D., Lipsitz, O., Rosenblat, J. D., ... Majeed, A. (2020). Antidepressant medications and weight change: a narrative review. Obesity, 28(11), 2064-2072.

Hirschfeld, R. M., Williams, J. B., Spitzer, R. L., Calabrese, J. R., Flynn, L., Keck Jr, P. E., ... Rapport, D. J. (2000). Development and validation of a screening instrument for bipolar spectrum disorder: the Mood Disorder Questionnaire. American journal of psychiatry, 157(11), 1873-1875.

jon, d.-i., yoon, b.-h., jung, h.-y., ha, k.-s., shin, y.-c., & bahk, w.-m. (2005). A validation study of the Korean version Mood Disorder Questionnaire (K-MDQ). Journal of Korean Neuropsychiatric Association, 44(5), 583-590.

Kay, S. R., Fiszbein, A., & Opler, L. A. (1987). The positive and negative syndrome scale (PANSS) for schizophrenia. Schizophrenia bulletin, 13(2), 261-276.

Kim, B.-Y., Kang, S. M., Kang, J.-H., Kang, S. Y., Kim, K. K., Kim, K.-B., ... Kim, J.-H. (2021). 2020 Korean Society for the Study of Obesity guidelines for the management of obesity in Korea. Journal of obesity & metabolic syndrome, 30(2), 81-92.

Kim, K.-K., Haam, J.-H., Kim, B. T., Kim, E. M., Park, J. H., Rhee, S. Y., ... Koo, H. Y. (2023). Evaluation and Treatment of Obesity and Its Comorbidities: 2022 Update of Clinical Practice Guidelines for Obesity by the Korean Society for the Study of Obesity. Journal of obesity & metabolic syndrome, 32(1), 1-24.

Mazereel, V., Detraux, J., Vancampfort, D., Van Winkel, R., & De Hert, M. (2020). Impact of psychotropic medication effects on obesity and the metabolic syndrome in people with serious mental illness. Frontiers in Endocrinology, 11, 573479.

Nicklas, J. M., Huskey, K. W., Davis, R. B., & Wee, C. C. (2012). Successful weight loss among obese US adults. American journal of preventive medicine, 42(5), 481-485.

Sabé, M., Pallis, K., Solmi, M., Crippa, A., Sentissi, O., & Kaiser, S. (2023). Comparative Effects of 11 Antipsychotics on Weight Gain and Metabolic Function in Patients With Acute Schizophrenia: A Dose-Response Meta-Analysis. The Journal of Clinical Psychiatry, 84(2), 45463.

Serretti, A., Mandelli, L., & Laura, M. (2010). Antidepressants and body weight: a comprehensive review and meta-analysis. The Journal of Clinical Psychiatry, 71(10), 1259-1272.

Stogios, N., Humber, B., Agarwal, S. M., & Hahn, M. (2023). Antipsychotic-Induced Weight Gain in Severe Mental Illness: Risk Factors and Special Considerations. Current Psychiatry Reports, 25(11), 707-721.

Zheng, P., Yu, Z., Mo, L., Zhang, Y., Lyu, C., Yu, Y., ... & Li, Y. (2022). An individualized medication model of sodium valproate for patients with bipolar disorder based on machine learning and deep learning techniques. *Frontiers in Pharmacology*, *13*, 890221.

Kang, Ji-Hyun, & Kim, Kyung-Gon. (2024). Practice guidelines for obesity clinics in primary care centers. *Journal of the Korean Medical Association*, *67*(4), 240-255.

Sung-Hoon Kim, Kyung-Min Kim, Bo-Hyun Yoon, Ha-Ran Jung, Jung-Ran Jeong, Hyun-Joo Yoon, ... Shi, Hwa-hwa. (2021). Long-term follow-up of severely mentally ill patients with metabolic syndrome: A 10-year follow-up study (2011-2020). Biotherapeutic Psychiatry, 27(1), 12-24.

American Society for Obesity. (2022). Obesity guideline 8th edition executive summary.

Jang, Jang-Won Son. (2022). State of the art of obesity pharmacotherapy. *Journal of Korean Diabetes*, *23*(2). 113-127.

Lee, J. H. (2014). Factors associated with long-term weight loss maintenance in obese patients. Ulsan National University Graduate School, Ulsan. (Master's thesis, Korea)

Dongseo Lee, Yongmin Ahn, Hyunkyun Shin, Seokkyun Ahn, Yeonho Joo, Seunghyun Kim, . . Lee, Ji-Yeon. (2001). Reliability and validity of the Korean version of the Positive and Negative Syndrome Scale. Neuropsychiatry, 40(6), 1090-1105.

Centers for Disease Control and Prevention. (December 8, 2023). National Health and Nutrition Examination Survey, ninth round, first year (2022). https://www.kdca.go.kr/board/board.es?mid=a20501010000&bid=0015&act=view&list_no=724014

Korea Health Promotion and Development Institute. (2021). Obesity statistics for national health promotion (2016-2020). (Project-02-2021-019-15).
