# Supplementary material for: Developing a machine learning algorithm to predict psychotropic drugs-induced weight gain and the effectiveness of anti-obesity drugs in patients with severe mental illness: Protocol for a prospective cohort study
Source: PLoS One. 2025 May 19;20(5):e0324000. doi: 10.1371/journal.pone.0324000 (PMC12088068; doi:10.1371/journal.pone.0324000)
Supplement: S3 File — (PDF) [file pone.0324000.s003.pdf]

정신약물의 체중증가 부작용 발생 가능성 및 비만치료제의 효과 예측 연구: 예비연구

Predicting the likelihood of weight gain as a side effect of psychiatric medications and the effectiveness of anti-obesity drugs: A pilot study

Version No 1.5

책임연구자 소속: 정신건강의학과

책임연구자 이름: 김선미

연구 개요

연구제목	(국문) 정신약물의 체중증가 부작용 발생 가능성 및 비만치료제의 효과 예측 연구: 예비연구
	(영문) Predicting the likelihood of weight gain as a side effect of psychiatric medications and the effectiveness of anti-obesity drugs : A pilot study
책임연구자	정신건강의학과 김선미
연구비 지원기관	중앙대학교

연구 목적	개개인에서 정신약물로 인한 체중증가와 대사 부작용의 발생을 예측하고, 정신약물로 인한 비만 발생 시 어떤 비만치료제가 효과적일지 예측하는 인공지능 알고리즘을 개발하는 것을 목적으로 함.
연구 설계	종적 조사 연구
연구 기간	IRB승인일 ~ 36개월
연구 대상<br>(시험약 등)	(1) 정신약물: 올란자핀(olanzapine), 퀘티아핀(quetiapine), 아리피프라졸(aripiprazole), 미르타자핀(mirtazapine), 발프로익산(valproate)<br>(2) 비만치료제: 오르리스타트(orlistat), 날트렉손/부프로피온 복합제(naltrexone/bupropion), 리라글루티드(liraglutide), 펜터민/토피라메이트 복합제(phentermine/topiramate), 펜터민(phentermine), 세마글루티드(semaglutide), 체중조절의 목적으로 처방되는 메트포르민(metformin), 토피라메이트(topiramate)
연구 대상자 수	300명
취약한 연구대상자	해당 없음
연구 방법	**[Phase 1] 정신약물의 체중증가 부작용 발생 가능성 연구**<br>우울증, 조울병, 조현병 환자를 모집하여 기저(baseline) 평가 시점에 인구학적 인자, 생활습관 인자, 가족력 및 동반이환 인자, 신체계측 및 체성분 인자, 혈액학적 인자, 심리척도 인자를 측정함. 주치의의 판단에 따라 올란자핀, 퀘티아핀, 아리피프라졸, 미르타자핀, 발프로익산 중 하나를 24주 동안 투여함. 12주째 신체계측 및 체성분 인자를 측정하고, 24주째 인구학적 인자를 제외한 모든 인자를 재측정함.<br>**[Phase 2] 정신약물로 인한 비만 발생 시 비만치료제의 효과 예측 연구**<br>Phase 1의 추적평가(24주) 시점에 체질량지수 25kg/m^2 이상인 비만 혹은 체질량지수 23kg/m^2 이상 25kg/m^2 미만인 비만전단계(과체중)에 해당하는 경우, Phase 2단계로 이행함. 대상자들은 정신약물 치료를 유지하며, 주치의의 판단에 따라 오르리스타트, 날트렉손/부프로피온 복합제, 리라글루티드, 펜터민/토피라메이트 복합제, 펜터민, 세마글루티드, 메트포르민, 혹은 토피라메이트 중 하나를 24주 동안 추가적으로 투여함(단,

	펜터민의 경우 최대 12주 동안 투여함). 비만치료제 추가 투여 12주 후에 신체계측 및 체성분 인자를 측정하고, 24주 후 인구학적 인자를 제외한 모든 인자를 재측정함(단, 펜터민의 경우에는 4주 후 신체계측 및 체성분 인자를 측정하고, 12주 이내에 투여 중단하며 사후 평가를 실시함)
유효성 평가	각각의 정신약물과 비만치료제, 그리고 두 약제의 조합이 체중과 비만·대사 관련 인자(인구학적 인자, 생활습관 인자, 가족력 및 동반이환 인자, 신체계측 및 체성분 인자, 혈액학적 인자, 심리척도 인자)에 일으키는 변화량을 딥러닝을 통해 학습시키고, 개인별 정신약물의 체중 증가 부작용 발생 가능성 및 비만치료제의 효과를 예측하는 인공지능 알고리즘을 개발함.
안전성 평가	이상반응 발생여부, 이상반응명, 중등도, 중대한 이상반응 여부 및 상세내용, 본제와의 인과관계, 이상반응으로 인한 약물 중단율
기대효과 및 예상결과	개개인에서 정신약물로 인한 체중증가와 대사 부작용의 발생을 예측하고, 정신약물로 인한 비만 발생 시 어떤 비만치료제가 효과적일지 예측하는 인공지능 알고리즘을 개발함.

연구계획서

1. 연구 제목

정신약물의 체중증가 부작용 발생 가능성 및 비만치료제의 효과 예측 연구: 예비연구

2. 연구의 실시기관 명칭 및 주소

중앙대학교병원, (06973) 서울특별시 동작구 흑석로 102 (흑석동 224-1)

3. 연구책임자 및 공동연구자 성명 및 직명

1) **연구책임자:** 김선미(부교수)
2) **공동연구자:** 한덕현(교수), 이혜준(임상조교수)
3) **연구담당자:** 김나연(임상강사), 김다슬(연구원)

4. **연구 의뢰기관**

1) **연구 의뢰기관 명칭 및 주소:** 해당 없음
2) **모니터요원 성명 및 직명**: 해당 없음

5. 연구비 지원기관 명칭 및 주소

중앙대학교, (06974) 서울특별시 동작구 흑석로 84

6. 예상연구기간

IRB 승인일로부터 ~ 36개월

7. 연구 대상 질환

우울증, 조울병, 조현병

8. 연구의 배경 및 목적

1) 연구 배경

비만은 가장 중요한 건강 문제 중 하나로 세계적인 비만 유행으로 인한 질병 부담 역시 가중되고 있음. 아시아인에서 비만은 체질량지수 25kg/m²이상으로 정의하며, 2022년 우리나라 성인의 비만 유병률은 37.2%임. 비만은 제2형 당뇨병, 고혈압, 이상지질혈증 등

의 대사증후군, 심뇌혈관질환, 암, 위장관 및 담낭 질환, 근골격질환 등 다양한 만성질환의 동반이환을 증가시키며, 사회적/정신적 장애 발생 및 사망 위험을 증가시킴. 따라서, 급속히 증가하는 비만으로 인한 여러 질환과 사망을 줄이기 위하여 비만 관리의 전략은 비만 치료는 물론, 체중 증가와 관련된 위험 요인의 적절한 예측과 관리, 예방으로 확대되어야 함.

전 세계적으로 볼 때, 조현병, 조울병, 중증 주요우울장애 등 중증 정신질환자의 비만 유병률은 25.9%이며, 과체중(비만전단계: 체질량지수 23-25kg/m²)까지 포함하면 그 유병률은 60.1%로 과반수 이상임. 일반 인구와 비교하여 중증 정신질환자가 비만일 가능성은 3.04배, 과체중 혹은 비만일 가능성은 2.03배 더 높음. 중증 정신질환자의 대사증후군 유병률 또한 26.1%로 일반 인구보다 발생 위험이 큼. 이들은 일반 인구 대비 사망률이 2-3배 높고 기대수명이 평균 10-20년 짧은 것으로 알려져 있으며, 주된 사망원인은 심혈관 질환임.

일부 항정신병약물, 항우울제, 기분조절제는 부작용으로 체중증가 및 대사 변화를 흔히 일으킨다고 알려짐. 국내 처방 상위 항정신병약물 중, 특히 올란자핀(olanzapine)과 쿠에티아핀(quetiapine)은 용량 의존적으로 체중증가와 더불어 다양한 대사관련 지표에 영향을 주는 것으로 나타남. 아리피프라졸(aripiprazole)은 작용기전 상 체중증가 부작용이 적을 것이라는 초기의 인식과 달리, 임상 경험이 쌓이면서 체중증가 부작용이 유의하게 보고됨. 최근 연구들을 통해서도 이 사실이 입증되면서 현재 많은 관심과 논의의 대상이 되고 있음. 항우울제의 체중증가 부작용에 대한 메타분석 연구에서, 최근 일차선택제로 쓰이지 않는 삼환계항우울제를 제외하고는 미르타자핀(mirtazapine)이 체중증가의 위험이 가장 큰 것으로 밝혀졌음. 또한, 가장 흔히 처방되는 기분조절제인 발프로익산(valproic acid)도 최대 50%의 환자에서 체중증가와 관련이 있다고 보고됨. 정신약물로 인한 체중증가는 대사증후군 및 심뇌혈관질환의 발생과 사망률 증가로 이어지며, 낮은 자존감, 삶의 질 저하, 의료비용 증가와 관련됨. 또한, 체중증가의 부작용은 치료순응도를 저해하는 대표적인 원인임.

현재 다양한 비만치료제들이 널리 처방되고 있으나, 개개인에서 이 중 어떤 비만치료제가 효과-부작용 측면에서 우세할지에 대한 연구는 부족함. 개별화된 비만치료제 처방이 아닌 획일적인 처방은 치료 효과를 저해하고, 비만치료제의 오남용 및 불필요한 의료비용 소모로 이어질 수 있음. 비만치료제의 효과 및 부작용은 유전적 요인, 사회경제적 요인, 생활습관, 체성분, 동반이환, 복용 중인 약물 등 다양한 인자에 따라 다르므로, 이를 포괄적으로 고려한 치료 효과 예측인자에 관한 연구가 필요함.

동일한 정신약물을 사용하더라도 체중증가에 대한 민감성은 개인마다 유의한 차이가

있으나, 아직 각각의 정신질환자에서 정신약물 치료 시 체중증가 정도를 예측할 수 있는 인자에 관한 연구는 부족함. 정신약물로 인한 체중증가 위험도는 개인의 유전적 요인, 생활습관, 체성분, 정신증상 심각도 등 다양한 요인과 복합적으로 관련되어 있으므로, 이를 포괄적으로 고려한 체중증가 예측인자에 관한 연구가 필요함. 최근 머신러닝을 활용하여 일반 성인에서 개인의 비만 위험도를 예측하는 연구들이 이루어지고 있으나, 아직까지 정신질환자에서 정신약물의 체중증가 위험도를 예측하는 주제의 머신러닝 연구는 보고되지 않음.

2) 연구 가설 및 목적

본 연구는 개개인에서 정신약물로 인한 체중증가와 대사 부작용의 발생을 예측하고, 정신약물로 인한 비만 발생 시 어떤 비만치료제가 효과적일지 예측하는 인공지능 알고리즘을 개발하는 것을 목적으로 함.

9. 임상연구용 의약품 및 (체외진단)의료기기 코드명(또는 주성분의 일반명), 원료약품의 분량, 제형 등(대조약 포함)

(1) 정신약물: 올란자핀(olanzapine), 쿼티아핀(quetiapine), 아리피프라졸(aripiprazole), 미르타자핀(mirtazapine), 발프로익산(valproate)

(2) 비만치료제: 오르리스타트(orlistat), 날트렉손/부프로피온 복합제(naltrexone/bupropion), 리라글루티드(liraglutide), 펜터민/토피라메이트 복합제(phentermine/topiramate), 펜터민(phentermine), 세마글루티드(semaglutide), 체중조절의 목적으로 처방되는 메트포르민(metformin), 토피라메이트(topiramate)

10. 연구대상자의 선정 기준, 제외기준, 목표한 대상자 수 및 산출 근거

[Phase 1] 정신약물의 체중증가 부작용 발생 가능성 연구

1) 선정기준

A. 만 19세 이상 성인

B. 정신질환에 대한 DSM-5 진단기준에 따라 우울증, 조울병 혹은 조현병으로 진단받은 자

C. 초기 평가 시점 기준 1개월 이내에 올란자핀(olanzapine), 쿼티아핀(quetiapine), 아리피프라졸(aripiprazole), 미르타자핀(mirtazapine), 발프로익산(valproate) 사용 과거력이 없는 자

D. 초기 평가 시점 기준 1개월 이내에 체중감소를 목적으로 한 비만치료제의 복용 이력이 없는 자

2) 제외기준

A. 현재 심각한 내과적 질환이 있는 자 (암, 심부전, 신부전, 간질환, 폐질환, 갑상선 질환, 급성 염증 상태 등)

B. 과거 또는 현재의 물질 의존이나 오남용 병력이 있는 자

C. 간질, 두부 외상, 기질적 정신 질환의 병력이 있는 자

[Phase 2] 정신약물로 인한 비만 발생 시 비만치료제의 효과 예측 연구

1) 선정기준

A. 본 연구의 Phase 1이 종료되는 시점에서 체질량지수 25kg/m^2 이상인 비만 혹은 체질량지수 23kg/m^2 이상 25kg/m^2 미만인 비만전단계(과체중)에 해당하는 자

2) 제외기준

Phase 1과 동일

3) 목표한 대상자 수

300명

4) 대상자 수 산출근거

양극성 장애 환자를 위한 발프로익산의 개별화된 약물치료 모델을 개발하기 위하여 머신러닝 및 딥러닝 기술을 이용한 기존 연구(Zheng, et al., 2022)에서 전자의무기록에서 최종 164건의 데이이터를 활용하여 73%~85%의 정확도를 보고함. 항정신병약물, 항우울제, 기분조절제의 체중증가 위험성을 예측하기 위해 머신러닝기술을 적용하여 66.05%~79.62%의 평균 정확도를 보고한 기존 연구(Eder, et al., 2024)에서도 163명에 환자가 포함되었으나 4주 후 탈락률이 37%로 높게 나타나 103명의 체중 변화가 확인됨. 이에 본 연구에서도 선행 연구와 유사한 수준의 데이터셋을 확보하기 위하여 약 40%의 탈락률을 고려하여 300명을 모집하고자 함.

5) 연구 대상자 모집 계획

본 연구는 중앙대학교병원 정신건강의학과에 모집 공고문을 게시하고, 외래/병동 환자를 대상으로 연구 안내문을 배포하여 연구 대상자를 모집함. 참여를 희망하는

대상자에게 연구에 대하여 설명하고 자발적으로 동의한 대상자에게 선정/제외 기준에 부합하는지 여부를 확인하여 연구를 진행함.

5) 연구 참여자의 동의 능력 평가

연구 참여자가 연구에 대해 충분히 이해하고 자발적으로 동의할 수 있는지를 평가하기 위해, 모든 참여자는 연구의 성격, 목적, 절차, 잠재적 위험 및 이점에 대해 충분히 이해한 후 동의하도록 평가됨.

(1) 평가절차: 연구자는 연구 내용을 이해하기 쉽게 설명하며 연구 목적, 절차, 잠재적 위험 및 이점, 그리고 언제든지 철회할 권리에 대해 열린 질문을 통해 참여자의 이해도를 확인함.

(2) 평가 기준: 참여자가 다음의 사항을 충족한 경우, 동의 능력이 있다고 판단함.

- 연구 정보에 대한 이해
- 본인의 결정을 명확히 전달할 수 있는 능력
- 본인의 가치와 선호에 부합하는 일관된 의사결정

11. 연구 방법

1) 구체적인 연구방법

- **[Phase 1]** 모집된 환자를 대상으로 기저(baseline) 평가 시점에 인구학적 인자, 생활습관 인자, 가족력 및 동반이환 인자, 신체계측 및 체성분 인자, 혈액학적 인자, 심리척도 인자를 측정함.
- 대상자가 기존에 복용하던 정신약물이 있다면 유지하는 상태에서, 유의한 체중증가 부작용이 있다고 알려진 올란자핀, 퀘티아핀, 아리피프라졸, 미르타자핀, 발프로익산 중 하나를 추가적으로 24주 동안 투여함.
- 정신약물 투여 12주 후에 신체계측 및 체성분 인자를 측정하고, 24주 후에 인구학적 인자를 제외한 모든 인자를 재측정함.

- **[Phase 2]** Phase 1의 추적평가(24주) 시점에 체질량지수 25kg/m^2 이상인 비만 혹은 체질량지수 23kg/m^2 이상 25kg/m^2 미만인 비만전단계(과체중)에 해당하는 경우, Phase 2 단계로 이행함. (Phase 2 선정 조건에 해당하는 대상자에게 연구 지속 참여 여부를 확인하여 이행을 결정함)
- 대상자들은 정신약물 치료를 유지하며, 주치의의 판단에 따라 오르리스타트,

날트렉손/부프로피온 복합제, 리라글루티드, 펜터민/토피라메이트 복합제, 펜터민, 세마글루티드, 메트포르민 혹은 토피라메이트 중 하나를 24주 동안 추가적으로 투여함. (단, 펜터민의 경우에는 최대 12주 동안 투여함)

- 비만치료제 추가 투여 12주 후에 신체계측 및 체성분 인자를 측정하고, 24주 후 인구학적 인자를 제외한 모든 인자를 재측정함. (단, 펜터민의 경우에는 예외적으로 4주 후 신체계측 및 체성분 인자를 측정하고, 12주 이내에 투여 중단하며 사후 평가를 실시함)

※ Study Flow

단계	Phase 1				Phase 2	
항목	Screening	Baseline	F/U	F/U	F/U	F/U
Visit	V0	V1	V2	V3	V4	V5
	-2wk~	D0	12wk±7d	24wk±7d	36wk±7d	48wk±7d
서면동의	●					
선정/제외 기준	●					
인구학적 정보	●	●				
약물처방		●	●	●	●	●
순응도조사			●	●	●	●
식이조사		●		●		●
식사태도검사(KEAT-26)		●		●		●
단문형 국제신체활동설문 (IPAQ)		●		●		●
위험음주자선별도구(AUDIT-K)		●		●		●
불면증 심각도 척도(ISI-K)		●		●		●
가족력 및 동반이환 조사		●		●		●
신체계측 및 체성분 조사		●	●	●	●	●
혈액 검사		●		●		●

양성 및 음성증후군 척도(PANSS)		●		●		●
벡 우울척도(BDI-II)		●		●		●
기분장애 질문지(MDQ)		●		●		●
* Visit 1과 Visit 2는 동일한 날에 시행할 수 있음.						

2) 비교군 설정 및 무작위 배정 방법

해당없음

3) 시험약 투여·사용량, 투여·사용 방법, 병용 요법, 대조약 사용시 그 선택사유

(1) 시험약:

- [Phase 1] 정신약물: 올란자핀, 퀘티아핀, 아리피프라졸, 미르타자핀, 발프로익산
- [Phase 2] 비만치료제: 오르리스타트, 날트렉손/부프로피온 복합제, 리라글루티드, 펜터민/토피라메이트 복합제, 펜터민, 세마글루티드, 체중 조절의 목적으로 처방되는 메트포르민, 혹은 토피라메이트

(2) 비만치료제 표준 진료 지침

- 대한비만학회 비만진료지침 8판(2022)에서 권고하는 약물치료의 지침은 다음과 같다. (1) 비만의 기본적인 비만의 기본적인 치료 방법은 식사치료, 운동치료 및 행동치료이며, 약물치료는 이들과 함께 시행하는 부가적인 치료방법으로 사용할 것을 권고한다. (2) 체질량지수가 25kg/m^2 이상이면서 비약물치료로 체중 감량에 실패한 환자에게 고려한다. (3) 장기간 체중 관리를 위해서는 대규모 임상 연구결과에 기초하여 사용 승인을 받은 약제를 사용할 것을 권고한다. (4) 비만치료제 유지 용량 투여 3개월 내에 5% 이상의 체중감량이 없다면 약제를 변경하거나 중단할 것을 권고한다.
- 비만 약물치료의 적응증은, 미국국립보건원에서는 BMI가 30kg/m^2 이상인 경우, 혹은 27kg/m^2 이상이면서 심혈관질환 고위험군(고혈압, 당뇨병, 이상지질혈증)이나 수면무호흡증이 동반된 경우 약물치료를 시도할 것을 권고하고 있다. 아시아-태평양 비만 치료 지침에서는 BMI가 25 kg/m^2 이상인 경우, 혹은 23 kg/m^2 이상이면서 심혈관질환 고위험인 경우 약물치료를 고려할 것을 제안하였다. 국내에서는 BMI

25 kg/m^2 이상인 환자에서 비약물치료로 체중감소에 실패한 경우에 약물치료를 고려하게 된다. 그러나 이러한 권고사항은 현재 미국국립보건원 기준과 동일한 국내 비만 약물 허가사항과 서로 상이하여, 약물 허가사항 외의 상황에서 약물투여를 하고자 하는 경우에는 환자와 충분히 상의하고 약물투여에 따른 이익과 손해에 대해 잘 설명한 후 환자의 동의를 얻고 시행해야 한다.

(3) 투여량 및 방법:

- 각 환자에게 처방되는 정신약물과 비만치료제의 종류와 용량은 약물치료 지침 및 의학적 판단에 따라 전문의가 결정하며 허용최대투여용량 내에서 조절한다.

※ 비만치료제의 처방

- 국내에서 비만치료제는 비급여로만 처방이 가능하며, 처방약, 투여 용량에 따라 차이가 있으나 일반적으로 월 20~40만원 정도의 비용이 발생한다.
- **펜터민**은 단기 비만치료제로 **4주 이내 투여**를 원칙으로 하고 4주 이내에 만족할 만한 체중 감량을 얻었을 경우 치료를 지속할 수 있으며, **총 처방 기간이 3개월**을 넘기지 않도록 한다. 통상 권장 용량은 15~37.5mg으로, 아침 식사 전이나 아침 식사 후 1~2시간 후에 복용한다.
- 장기사용 비만치료제의 경우 치료 기간은 개인차가 있으나 보통 6~10개월 정도 실시하는 것이 일반적이다.
- **오르리스타트**는 장기 체중 관리 허가를 받은 비만 치료제로 권장용량은 **1일 3회, 1회 120mg**이다. 오르리스타트는 식사 전 120mg 복용 시 식이 지방의 약 30% 흡수를 방지하며, 더 높은 용량은 추가 효과가 없다.
- **날트렉손/부프로피온은** 장기 사용 가능한 비만치료제로 승인을 받았다. 권장되는 투여 방법은 **첫 주 아침 8/90mg 1정, 둘째 주 아침 1정 저녁 1정, 셋째 주 아침 2정 저녁 1정, 넷째 주 이후 아침 2정 저녁 2정**의 순서로 4주에 걸쳐 천천히 1정씩 증량한 뒤 환자에게 적절한 용량으로 유지하는 것이다. (1일 최대 용량: 날트렉손 32mg, 부프로피온 360mg)
- **펜터민/토피라메이트는** 장기적인 체중 관리에 대해 승인된 약물로, 주요 사용하는 유지 용량은 7.5/46mg 혹은 15/92mg 1일 1회 투여하는 것이다. 내약성을 높이기 위해 처음 **14일 동안은 1일 1회 3.75/23mg**으로 시작하고 그 후 **12주 동안 7.5/46mg으로 증량**하여 투여를 유지한다. 12주 후에도 기준 체중이 3% 이상 감소하지 않은 경우 **14일 동안 11.25/69mg으로 증량**한 다음 **15/92mg으로 증량할**

수 있다. 매일 아침 음식과 함께 또는 없이 복용하며, 불면증을 피하기 위해 저녁에는 복용하지 않도록 한다.

- **리라글루티드**는 **피하주사 방식으로 1일 1회** 체내에 투여하며, 초기 용량은 일주일 동안 **0.6mg 투여 후 일주일 간격으로 0.6mg씩 단계적으로 증량**한다. 최대 투여 용량은 3.0mg으로 이후 유지한다.
- 체중 감량 보조제로서 **메트포르민**의 용량 범위는 확립되지 않았으나, 보통 하루 500mg에서 2,000 mg가 사용된다. 당뇨병 치료 최대 용량은 매일 3회 850mg이며, 환자들은 저용량으로 시작해 약물의 효과를 확인해볼 수 있다. **1일 2-3회 1회 500mg** 용량으로 시작하여, 용량 증가는 **매주 500mg씩 점차적으로 증량**한다.
- **토피라메이트**는 저용량으로 시작하여 식사와 관계없이 1일 1회 복용한다. 토피라메이트는 한 달 이상 100~200 mg/day 용량으로 복용했을 때 체중 감량 효과가 크며, 25 mg/day의 시작 용량에서도 체중 감량이 나타난다. 통상 첫 1주간 저녁 25mg을 투여한 후, 1-2주 간격으로 1일 25mg 또는 50mg씩 증량한다. 1일 권장량은 100-200mg이며, 1일 최대 용량은 500mg이다.
- **세마글루티드**는 주 1회 피하주사로 투여된다. 초기 투여 용량은 주 1회 0.25mg으로 시작하여, 약 4주마다 점진적으로 증량하여 최종적으로 주당 2.4mg의 유지 용량에 도달한다.

※ 동반질환에 따른 비만치료제의 선택

표 1. 동반 질환에 따른 항비만약제의 선택

동반 질환		항비만약제			
		Orlistat	Naltrexone / bupropion ER	Liraglutide 3.0 mg	Phentermine/ topiramate ER
2형 당뇨병					
고혈압			혈압,맥박 관찰<br>조절되지 않는 고혈압의 경우 금기	맥박 관찰	맥박 관찰
관상동맥 질환			혈압, 맥박 관찰		
만성 신질환	경증(60-89 mL/min)				
	중등도(30-59 mL/min)		8 mg/90 mg bid /day 초과하지 않음		7.5 mg/46 mg/day 초과하지 않음
	중증 (<30 mL/min)	요로결석(oxalate) 주의	8 mg/90 mg bid /day 초과하지 않음<br>말기 신장애 권고되지 않음		7.5 mg/46 mg/day 초과하지 않음<br>말기 신장애 권고되지 않음
간기능 장애	경도~중등도 (Child-Pugh 5-9)	담석 주의	8 mg/90 mg/day 초과하지 않음	담석 주의	7.5 mg/46 mg/day 초과하지 않음
	중증(Child-Pugh >9)				
폐쇄성 수면무호흡					15 mg/92 mg/day 사용
우울증					7.5 mg/46 mg/day 초과하지 않음
녹내장					
췌장염					

사용 가능 주의 사용 권고되지 않거나 사용 금기 근거부족

출처: 대한비만학회, 비만 치료 지침 요약본 8판 (2022).

4) 관찰항목, 임상검사항목 및 관찰검사방법

㉠ 인구학적 인자: 성별, 연령, 교육수준, 소득수준, 결혼 여부, 직업 등

㉡ 생활습관 인자: 식이(영양), 운동(신체활동), 음주, 수면 등

- 식이: 최근 1주일 중 3일 동안 평소의 식사와 유사한 날의 식사를 기록하도록 하여, 총 에너지 및 영양소 섭취량 계산, 한국판 식사태도검사(The Korean Version of Eating Attitudes Test-26; KEAT-26, 26문항)를 이용하여 식사 관련 문제 평가
- 운동: 한국형 단문형 국제신체활동설문(International Physical Activity Questionnaire; IPAQ, 7문항)을 이용하여 신체활동 평가

- 음주: 한국어판 위험음주자 선별도구(Alcohol Use Disorder Identification Test; AUDIT-K, 10문항)를 이용하여 음주 평가

- 수면: 한국판 불면증 심각도 척도(Insomnia Severity Index; ISI-K, 5문항)를 이용하여 수면 평가

ⓒ 가족력 및 동반이환 인자: 비만의 가족력, 동반이환(대사증후군 포함), 비만치료제 복용 이력

ⓓ 신체계측 및 체성분 인자: 키, 허리둘레, 허리둘레/엉덩이둘레 비율 측정. 생체전기저항분석(bioelectic impedanceanalysis, BIA)을 이용하여 체중, 체질량지수, 체지방량, 제지방량, 근육량, 복부지방률 등 측정

ⓔ 혈액학적 인자: 인슐린, 공복혈당, 지질(총콜레스테롤, 저밀도지단백콜레스테롤, 고밀도지단백콜레스테롤, 중성지방), 요산, 고감도 C-반응단백 등 측정

ⓕ 심리척도 인자: 조현병 증상 점수, 우울증 점수, 조울증 척도 점수

- 양성 및 음성증후군 척도(Positive and Negative Syndrome Scale; PANSS, 30문항): 구조화된 면담을 통해 조현병 양성 증상과 음상 증상을 평가

- 벡 우울척도 2판(Beck Depression Inventory–II; BDI-II, 21문항): 우울 증상을 평가하는 가장 보편적인 척도

- 기분장애 질문지(Mood Disorder Questionnaire; MDQ, 15문항): 양극성 스펙트럼 장애 선별 척도로 임상에서 증상 심각도 평가에 활용

ⓢ 심전도 검사, 임신반응검사: [Phase 2]에서 비만치료제 처방 전, 금기사항을 확인하기 위해 필요할 경우에만 실시

5) 유효성 평가기준, 평가방법

개개인에서 정신약물로 인한 체중증가와 대사 부작용의 발생을 예측하고, 정신약물로 인한 비만 발생 시 어떤 비만치료제가 효과적일지 예측하는 인공지능 알고리즘 개발 여부

6) 기존 치료 및 연구와의 차별점

최근 머신러닝을 활용하여 일반 성인에서 개인의 비만 위험도를 예측하는 연구들이 이루어지고 있으나, 아직까지 정신질환자에서 정신약물의 체중증가 위험도를 예측하는 주제의 머신러닝 연구는 보고되지 않음. 이를 의학적 의사결정에 참고하여 정신약물 치료계획 수립 당시부터 최적의 정신약물을 선택할 수 있다면, 환자의 정신 증상 개선과 더불어 비

만 및 대사증후군을 예방함으로써 신체 건강도 효과적으로 관리할 수 있을 것으로 기대됨. 이는 다시 장기적인 치료순응도를 향상함으로써 정신질환 증상 관리에도 유리할 것임. 또한, 비만으로 진단된 정신질환자에게 최적의 비만치료제를 선택할 수 있다면, 비만 및 대사증후군을 신속하게 치료하여 합병증을 예방하고, 정신약물치료에 대한 장기적인 치료순응도를 향상함으로써 결과적으로는 정신질환 증상 관리에도 유리할 것으로 예상됨.

7) 연구대상자의 이익과 위험

(1) 이익: 본 연구 참여에 따른 직접적인 이익은 없음. 다만, 매 측정시점에 따른 검사 및 평가 결과를 통해 건강 상태의 변화를 확인할 수 있으며, 본 연구 참여로 조사된 사항에 대한 결과를 제공받을 수 있음. 본 연구의 결과는 정신약물의 체중증가 부작용 발생 가능성 및 비만치료제의 효과를 예측하는 알고리즘을 개발하는데 기여하게 될 것임.

(2) 위험: 본 연구에서 사용하는 정신약물은 일반적으로 체중증가의 부작용이 보고되고 있음. 비만치료제 중 **펜터민**과 같이 단기 사용 승인받은 식욕억제제는 교감신경을 활성화시키기 때문에 현기증, 불면증, 불안감, 입마름 등이 나타날 수 있고, 혈압 상승, 빈맥, 심계항진, 원발성 폐동맥 고혈압, 역류성 심장판막 질환 등 심혈관계 부작용이 나타날 수 있음. **오르리스타트**는 변실금 및 지방변, 복부팽만과 같은 소화기계 부작용이 자주 나타날 수 있으며, 드물게 간 손상이 보고된 바 있음. **리라글루티드**의 흔한 부작용은 위장관계 증상(구토, 설사, 변비, 소화불량, 복통), 담낭 관련 증상(담석, 담낭염)임. 췌장염 과거력이 있거나 갑상선 수질암과 다발성 내분비 선종증 과거력 또는 가족력이 있는 경우 금기임. **날트렉손/부프로피온**의 가장 일반적인 부작용은 메스꺼움, 구토, 변비, 두통, 어지러움, 수면장애, 입마름 등임. 항정신병용제나 항우울제를 복용 중인 기분장애 및 정신질환 환자의 경우 약물 상호작용과 발작 위험의 증가로 처방 시 주의가 필요함. 노르에피네프린 재흡수 억제제(SNRI)와 함께 부프로피온을 복용하는 환자에게 세로토닌 증후군, 섬망, 초조, 불면증, 심박수 및 혈압상승, 경조증, 자살 충동의 발생이 보고된 바 있고, 세로토닌 재흡수 억제제(SSRI)와 부프로피온을 함께 복용하는 환자에게서 세로토닌 증후군, 발작, 섬망, 불면증, 현기증 및 위장관 이상반응의 발생이 보고된 바 있음. 양극성장애, 알코올, 벤조디아제핀, 바르비탈류, 항간질약 등 약물복용을 갑자기 중단한 자, 대식증, 신경성 식욕부진 환자에게는 투여 금기임. **펜터민/토피라메이트**는 일반적으로 이상감각, 현기증, 구강건조, 변비, 미각상실, 불면증, 불안 등의 부작용

이 보고되고 있음. 급격하게 중단하는 경우 발작 증세를 유발할 수 있으므로 점진적으로 중단해야 함. **토피라메이트**는 집중력/주의력 장애, 어지러움, 흥분, 감정불안, 우울증과 같은 정신신경계 이상반응이 보고되고 있음. 토피라메이트를 포함한 항경련제는 자살 충동을 증가시킬 수 있으므로 우울증의 발생 및 악화, 자살 충동, 환자의 기분이나 행동의 변화에 대해 모니터링 해야함. 또한, 토피라메이트는 리튬, 발프로익산, 발프로에이트세미소디움의 독성을 증가시킬 수 있어 1등급 심각한 부작용으로 보고되고 있으므로 병용 투여하지 않도록 유의해야 함. **메트포르민**은 유산증(증상: 무력감, 근육통, 복부풀쾌감, 호흡곤란), 저혈당증 일으킬 수 있으니 주의해야 함. 일반적으로 오심, 구토, 복부팽만, 식욕부진, 소화불량, 변비, 복통 등의 소화장애를 일으킬 수 있으며, 피부발진, 비타민 B12 결핍의 부작용이 발생할 수 있음. **세마글루티드**는 당뇨병성 망막변증 합병증이 흔한 빈도로 보고되어 있으며, 두통, 구토, 복통, 설사 등의 증상과 저혈당 위험이 흔하게 나타날 수 있음. 드물게 급성 췌장염과 저혈압이 나타날 수 있음. 세마글루티드와 정신과 약물과의 상호작용은 보고된 바 없으며 병용 시 안전성에 문제가 없는 것으로 판단됨. 다만, 세마글루티드는 위 배출 시간을 지연시켜 경구용 약물의 흡수에 영향을 미칠 수 있음.
이 외에도 각종 검사에 따른 시간 소요의 불편감이 있을 수 있고, 혈액을 채취하는 과정에서 통증, 멍, 머리가 아찔한 느낌이 동반될 수 있음. 환자가 이러한 불편감을 경험한다면 연구책임자 또는 공동 연구자의 정밀 문진을 통해 차후 대처방안을 모색할 것임. 또한, 연구대상자의 위험과 불편함을 최소화하기 위해 연구진은 항상 주의를 기울이며 필요한 경우 의학적 처치를 받도록 할 것임.

※ 비만치료제 별 이상반응, 주의사항, 투여금지 대상

기간	분류	약물(상품예)	이상반응	주의	투여금지
단기 치료제	교감 신경 흥분제	Phentermine (푸리민정)	• 입마름, 감각이상, 변비, 어지러움, 시야흐림, 안통, 불면증	• 최근 1년 내 다른 식욕억제제 사용 환자에게 투여 권장 않음<br>• 갑자기 중단 시 극도의 피로, 우울증, 수면, ECG 변화 초래<br>• 경구 당뇨약(투여량 조절)<br>• 늦은 밤 복용 시 불면 유발 가능	• 16세 이하 X<br>• 임부(DUR금기 1등급), 수유부<br>• 다른 식욕억제제 병용 X<br>• 심혈관, 뇌혈관환자<br>• 고혈압(폐동맥·중등도 이상)<br>• 신, 간장애(중등도 이상)

					• 중증 심·췌장 장애<br>• 녹내장<br>• 약물, 알코올 남용 이력<br>• MAOIs(14일 이내)<br>• 동맥 폐색 환자<br>• 갑상샘기능 항진증<br>• 정신적으로 매우 불안, 흥분상태
장기 치료제	리파제 저해제	Orlistat<br>(제니칼 캡슐)<br>12세 이상<br>(FDA)	• 복부팽만, 방귀, 지방변	• 지용성비타민 흡수 감소 (종합비타민 권고: 2시간 간격)<br>• Cyclosporine: 3시간 간격	• DUR 임부금기 1등급<br>• 만성 영양실조<br>• 담낭질환
	GLP-1 수용체	Liraglutide<br>(삭센다펜주)<br>12세 이상	• 오심, 구토, 설사, 변비, 복통, 두통, 어지러움, 피로<br>• 저혈당, 탈수<br>• 담낭염, 췌장염	• 자살위험 증가<br>• 급성 담관질환, 혈압 증가<br>• 인슐린 대용으로 사용말 것<br>• Sulfonylurea 병용 시 저혈당 위험 증가<br>• 와파린: 자주 INR 모니터링	• DUR 임부금기 2등급<br>• 본인 또는 가족의 갑상선암(MTC, MEN2)
	복합제	Phentermine/<br>topiramate<br>(큐시미아캡슐)<br>18세 이상	• 미각/감각 이상, 저림, 현훈, 불안, 구강 건조, 불면	• 태아 독성(임신테스트 음성 확인 후 처방)<br>• 중단 시 서서히 중단(경련 위험 ↓)<br>• 저혈당증, 도핑 리스트<br>•병용금기 (phendimetrazine, mazindol, selegiline, bupropion, naltrexone, diethylpropion)	• DUR 임부금기 1등급, 수유부<br>• 관상동맥 질환<br>• 조절되지 않는 고혈압<br>• 녹내장<br>• MAOIs(14일 이내)<br>• 갑상선 기능 항진증<br>• 정신적으로 매우 흥분, 불안상태
		Naltrexone/<br>bupropion<br>(콘트라브 서방정)<br>성인 ~ 75세 미만	• 오심, 구토, 변비, 두통, 현훈, 불면, 구강건조<br>• 혈압상승, 빈맥, 안압상승, 시야결손	• 자살충동, 혈압, 심박수↑<br>• 경련위험 증가<br>• 폐쇄각 녹내장 유발 가능<br>• 간독성<br>• 신결석예방(충분한 물섭취)	• DUR 임부금기 1등급<br>• 수유부, 뇌전증<br>• 간장애, 말기 신장애<br>• 양극성장애, 마약의존자<br>• 조절되지 않는 고혈압<br>• MAOIs(14일 이내)<br>• 알코올, BZ, Barbital, 항간질약 갑자기 중단 시

					• 대식증, 신경성 식욕부진 환자

출처: 비만 치료의 최신 지견- 대한비만학회 2022 진료 지침을 중심으로 (2023), 약학정보원 팜리뷰

8) 중지·탈락 기준

다음과 같은 상황이 발생할 경우 그 즉시 연구참여자 참여가 중단됨.

① 연구대상자가 참여 거부 의사를 밝혔을 시

② 연구 진행 도중 의료진과 상의 없이 임의로 2주 이상 처방약물 복용을 중단한 경우

③ 임상 시험 도중 대상자에서 제외될 만한 임상적 소견 발견 시

④ 기타 부득이한 사정으로 더 이상 임상 시험에 참여할 수 없게 되었을 시

9) 부작용을 포함한 안전성의 평가기준, 평가 방법 및 보고 방법

▶ 안전성 관련 용어의 정의

① 이상반응 (Adverse Event, AE): 시험약품 사용 후 나타나는 바람직하지 않고 의도되지 않은 증후 (Sign), 증상 (Symptom) 또는 질병을 말하며, 해당 시험약품과 반드시 인과관계를 가져야 하는 것은 아님.

② 중대한 이상반응 (Serious AE)

시험약품의 임의의 용량에서 발생한 이상반응 중에서 다음 중 하나에 해당하는 경우

- 연구대상자의 사망
- 생명을 위협하는 경우(연구대상자가 그 사건의 발생시점에서 죽음의 위협에 놓여 있는 경우)
- 지속적 또는 의미 있는 불구나 기능 저하를 초래하는 경우
- 입원을 요하거나 이미 입원한 환자의 입원 기일을 늘려야 하는 경우
- 선천적 기형 또는 이상을 초래하는 경우
- 기타 의학적으로 중요한 상황

위에서 열거한 상황이 아니더라도 의학적으로 연구대상자의 안위와 건강상태에 중대한 영향을 미칠 것으로 사료되는 상황이 발생한 경우, 관련 전문가의 의학적 판단에 따라 중대한 이상반응으로 간주할 것인가의 여부를 결정하고 이에 따라 적절한 조치를 취함.

- 품질불만정보 (PQC)

의료전문가, 소비자, 영업사원, 규제당국, 제휴/협업 회사, 지사 또는 다른 제3자에 의해 제공된 정보로서, 의약품 혹은 의심되는 위조 의약품의 확인(identify), 품질(quality), 내구성(durability), 신뢰성(reliability), 안전성(safety), 효능(effectiveness) 또는 성능(performance)과 관련된 결함 정보를 말함. PQC는 안전성정보를 포함할 수 있지만 이에 한정되지 않음.

▶ 평가방법

- 시험의약품 투약 시점부터 투약 종료 후 28일까지 발생한 모든 안전성 정보는 이상반응조사서에 빠짐없이 기록되어야 하며, 기록되지 않은 것은 주관적인 증상으로 분류됨.
- 이상반응 정도에 대한 평가는 연구담당자가 평가기준을 참고하여 증상의 경중에 따라 단계별로 평가하는 것을 원칙으로 함.

▶ 평가 기준

* 이상반응의 정도

- 경증(mild): 쉽게 견딜 수 있는 이상반응
- 중등도(moderate): 일상 생활에 상당한 지장을 주는 이상반응
- 중증(severe): 정상적인 일상 생활을 할 수 없을 정도의 이상반응

* 경과

- 완전 치유(후유증 없음)
- 치유(후유증 있음)
- 진행 중
- 영구적 손상
- 사망

* 임상연구용 의약품에 대한 조치

- 일시적 중지(Drug interrupted)
- 투여 중단(Drug withdrawn)
- 감량(Dose reduced)
- 증량(Dose increased)
- 해당사항 없음(Not applicable)

▶ 시험약품과의 인과관계

이상반응 발현 시 시험약품과의 연관성 여부는 연구책임자가 다음과 같이 분류하고 필요 시 연구담당자의 견해를 부기함.

- 명확히 관련 있음(Definitely related)
- 관련 있다고 생각됨(Probably related)
- 관련 있을 가능성 있음(Possibly related)
- 관련이 없다고 생각됨(probably not related)
- 명확히 관련이 없음(Definitely not related)
- 불명(Unknown)

▶ 보고방법

① 이상반응의 보고

- 연구책임자는 연구담당자 및 연구대상자 또는 보호자에게 약물 섭취 후 나타날 수 있는 모든 이상반응에 대하여 교육을 실시하고 섭취 후 나타나는 모든 현상에 대하여 보고하도록 교육을 실시함.
- 약물 사용 후 전신적 또는 임상병리학적으로 나타나는 제반 증상에 대하여 종류, 발생시간, 정도, 처치, 치료약, 경과, 시험약품과의 인과관계 등에 대하여 증례기록서에 기입함.
- 연구책임자는 연구 결과보고 시 연구 기간 중 발생된 모든 증상에 대하여 서술하고 평가를 실시하며, 연구기간 중 "중대한 이상반응 (Serious adverse event)" 발생시에는 기관생명윤리위원회 (IRB)에 보고하여 연구의 지속 또는 중단 여부를 결정함.
- 추가적인 안전성 정보를 주기적으로 해당 이상반응이 종결(해당되는 이상반응의 소실 또는 추적조사의 불가 등)될 때까지 보고하여야 함.
- 연구책임자는 임상시험을 시행함에 있어서 모든 제반 사항을 헬싱키 선언에 준하여 시행함.

② 신속 보고

- 목적

연구자 및 기타 관계자로 하여금 중대한 이상반응에 대한 새롭고 중요한 정보를 알게 하기 위함.

- 보고 대상

중대한 이상반응, 예상하지 못한 이상반응, 일반적으로 시험약품의 위험·편익 평가에 영향을 줄 수 있는 정보(예를 들어 새로 시행된 동물실험에서 중요한 안전성 정보를 알게 된 경우), 시험·대조약품 섭취에 변화를 줄 수 있는 정보 등이 이에 해당함.

- 보고 시기

해당 사안이 발생한 경우 연구책임자는 기관생명윤리위원회(IRB)에서 규정한 보고 기한 내에 보고하여 연구의 지속 또는 중단 여부를 결정함.

- 보고 방법

중대한 이상반응의 신속 보고 시에는 기관생명윤리위원회(IRB)의 중대한 이상반응 보고서 양식을 이용하며, 신속보고 시점에서 모든 정보를 얻을 수는 없더라도 가능한 많은 정보를 얻기 위해 최대한 노력을 기울여야 함.

▶ 중대한 이상반응 및 품질불만정보 발생시 조치사항

본 연구기간 중 연구책임자 및 담당자는 연구대상자의 안전에 만전을 기하여야 하며, 중대한 이상반응 및 품질불만정보 발생시에는 신속하고 적절한 조치를 취하여 중대한 이상반응을 최소화하여야 함.

임상시험 중 각 담당자의 의무는 다음과 같음.

① 연구책임자의 의무

시험자가 인지한 시점에 기관생명윤리위원회(IRB)에 보고하며, 보고 시점은 기관 규정에 따름. 필요 시 해당 시험약품에 대한 임상시험의 일부 또는 전부를 중지하여야 함.

② 연구담당자의 의무

시험자가 알게 된 시점으로부터 24시간 이내에 연구책임자 및 의뢰자에게 보고해야 함.

③ 기관생명윤리위원회(IRB)의 의무

중대한 이상반응을 보고 받은 후 임상시험의 일부 또는 전부에 대하여 중지명령 등 필요한 조치를 연구책임자에게 하여야 함.

④ 의뢰자의 의무

복수의 실시기관에서 임상시험을 실시하는 경우 중대한 이상반응이 발생한 실시기관으로부터 보고받은 내용을 타 실시기관에 즉시 통보하여야 함.

*자살 위험성 모니터링

시험자는 자살 위험성에 대한 모니터링을 고려하여야 함.

10) 자료안전성 모니터링 계획(DSMP)

- 본 연구는 기 판매되고 있는 약의 허가된 용량을 적용하며 이상반응의 빈도가 높지 않은 약물이므로 Level II (최소위험에서 약간 증가)에 해당됨.

- 모니터링 책임자: 정신건강의학과 부교수 김선미
- 모니터링 빈도: 환자를 모집하거나 추적 경과를 보는 기간 동안에는 최소 6개월에 1회 이상
- 본 기관에서 중대 이상 반응이 발생하지 않는 경우는 모니터링 결과를 최종 보고시에 같이 보고함 (안전성 관련 정보 보고서).
- 만약 중대한 이상 반응이 발생하는 경우 그 내용에 대해 "이상 약물/의료기기 반응 보고서(본원 또는 본원 외 국내/해외용)"를 피험자보호센터 일반적인 기준에 준하여 보고함
- 기관에서 사망 또는 생명위협에 해당하는 중대한 이상 반응이 1건이라도 보고되거나, 기타 중대 이상 반응이 거의 확실함 또는 확실함의 인과 관계로 3회 이상 발생시 책임연구자는 공동연구자들과 연구 조기 종료를 상의하고 그 결과를 피험자보호센터에 보고함.

11) 자료 분석 및 통계 분석 방법

각각의 정신약물과 비만치료제와의 조합이 체중과 비만·대사 관련 인자에 일으키는 변화량을 딥러닝을 통해 학습시키고, 개인별 정신약물의 체중 증가 부작용 발생 가능성 및 비만치료제 효과를 예측하는 인공지능 알고리즘을 개발하고자 함.

▷ 딥러닝 모델 개발 프로세스
- 중앙대학교 공과대학 AI 대학원에서 딥러닝 모델과 AI 알고리즘의 개발을 담당하기로 함.

㉠ 데이터 증강
- 적은 양의 데이터를 위한 데이터 증강 기법의 적용
  · SimSiam 같은 대조학습과 함께 가중치를 적용하여 다양한 뷰로 증강 방법
  · Variational Autoencoder를 통해 표 형식 데이터를 인위적으로 늘리는 증강 방법

㉡ 딥러닝 기반 모델 설계
- Tree 기반 방식의 필요성: 모델 결정 방식과 추론 결과에 대한 해석 용이, 빠른 학습과 추론 속도
- Transformer 기반 방식의 필요성: 비선형 관계 및 고차원 데이터에 대한 복잡한 패턴 학습 용이, 이미지, 텍스트, 음성 등 다양한 유형의 데이터를 결합하여 처리 가능
- 두 방식의 장점을 통합하는 하이브리드 모델 설계

· Tree 기반 모델과 Transformer 기반 모델의 장점을 결합한 정형 데이터 특화 모델 설계(TabNet, TabLLM 등)

㉢ 딥러닝 기반 모델 학습 및 평가

- 각 인자의 시계열 특성을 고려하기 위한 Attention 메커니즘 적용
- 모델 일반화 성능 및 데이터 효율성 향상을 위한 Model-Agnostic Meta-Learning 기반의 학습
- 다양한 인자들의 변화량에 대하여 RMSE 등의 지표를 활용한 모델 성능 평가

12) 연구수행일정표

IRB 승인일 ~ 30개월: 대상자 모집, 모집된 대상자에게 연구자료 수집

31개월 ~ 36개월: 데이터 전처리, 딥러닝 모델 및 AI 알고리즘 개발

12. 연구대상자의 안전보호를 위한 대책

1) 연구의 윤리성 확보를 위한 기본 방안

- 참여하는 모든 연구자들은 임상윤리교육을 모두 최근 2년내에 이수하였으며 교육내용에 따라 연구를 시행하며 연구책임자는 생명윤리 및 안정에 관한 법률을 포함한 국내법과 헬싱키 선언 등 국제 지침을 준수하며 연구대상자의 존엄성, 권리, 안전 및 안녕을 존중하고 연구의 결과가 전 세계의 모든 인류에 혜택이 될 수 있도록 최선의 노력을 다할 것임.
- 대상자에게 자세한 연구 설명 후 연구정보가 포함되어 있는 동의서 복사본을 제공함.
- 검사 시행 후 이상소견이 발견될 경우 지체 없이 대상자에게 알릴 것이며 적절한 정보 및 치료를 받을 수 있도록 최선을 다할 것임.

2) 연구대상자의 동의 과정

· 연구대상자에게 설명하고 동의를 취득할 연구자: 정신건강의학과 부교수 김선미, 정신건강의학과 교수 한덕현, 가정의학과 임상조교수 이혜준, 정신건강의학과 임상강사 김나연

· 동의를 제공할 자: 연구대상자

· 연구 설명 과정과 동의 취득 과정 사이의 대기 시간: 만일 연구대상자가 연구 설명 12시간 이후에 동의 의사를 밝힌 경우 연구대상자와 대리인에게 연구를 재설

명함.

· 강제 또는 부당한 영향의 가능성을 최소화시킬 방법: 모든 연구 참여와 관련된 내용 설명 시 연구대상자 또는 대리인의 동의가 필수이며, 모집된 이후에도 담당 연구원이 연구 내용에 대해 세부적 사항까지 다시 안내하여 대상자가 참여 내용에 대한 파악 없이 연구가 진행되는 일이 없도록 함.
· 연구 설명 과정과 동의 취득 과정에서 연구자가 사용하는 언어: 한국어
· 연구대상자 또는 대리인이 이해할 수 있는 언어: 한국어
· 연구대상자 또는 대리인에게 제공되는 정보와 동의서 서식: 연구 설명문 및 동의서 참고

3) 연구대상자의 보상 방안

Phase 1 기저평가 완료 시 2만원, 24주 후 추적 평가 완료 시 2만원의 교통비를 지급함. Phase 2로 이행하여 24주 후(기저평가 48주 후) 추적 평가를 완료하였을 시에는 2만원의 교통비를 추가로 지급함. 즉, phase 1에서 종료되는 연구대상자는 총 40,000원, phase 2에서 종료되는 연구대상자는 총 60,000원의 교통비를 지급받음. 검사 비용은 중앙대학교에서 연구비를 지원받아 연구자가 부담함.

4) 연구대상자의 개인정보보호 방안

본 연구 참여 과정에서 연구대상자에게 수집하는 자료는 대상자의 인구학적 정보(성별, 연령, 교육수준, 소득수준, 결혼 여부, 직업 등), 생활습관(식이, 운동, 음주, 흡연), 가족력 및 동반이환, 신체계측 및 체성분 정보, 혈액검사 결과, 임상 척도 문항에 대한 응답 등임.

또한, 연구대상자 모집 및 보상 지급 시 연구대상자의 전화번호, 계좌번호 등이 수집되며, 이는 보상 지급 후 즉시 폐기함. 수집된 의료기록, 검사결과, 건강정보, 정보분석을 통한 연구 결과는 연구를 감독하는 정부기관과 연구비 지원기관, 병원 내 생명윤리위원회 등이 관계 법령에 따라 임상시험의 절차와 자료의 품질을 검증하기 위하여 열람하거나 다른 관련된 연구 및 출판물에 인용될 수 있음. 다만, 환자의 신원을 파악할 수 있는 기록은 비밀로 보장하며 건강 정보는 성명을 가린 채로 제공함.

대상자가 연구 참여 동의 철회 시 법이 허용하는 범위 내에서 대상자가 연구에 참여한 시점까지 수집된 정보를 활용할 수 있음. 생명윤리법 시행규칙 제15조에 따라 연구 관련 기록을 연구가 종료된 시점부터 3년간 중앙대학교병원 정신건강의학과 연

구실에서 보관할 것이며, 보관 캐비닛은 잠금 장치로 보안을 유지하고 관련자 소수에게만 열쇠를 부여하여 접근 가능하도록 함. 보관기관이 지난 문서 중 개인정보 및 민감정보에 관한 사항은 개인정보보호법 시행령 제16조에 따라 파기함.

5) 취약한 연구대상자를 포함하는 경우 추가적인 보호조치 방안

해당 없음

13. 인체유래물의 보관 및 폐기 방법

해당 없음

14. 참고 문헌

Afzal, M., Siddiqi, N., Ahmad, B., Afsheen, N., Aslam, F., Ali, A., . . . Khalid, H. (2021). Prevalence of overweight and obesity in people with severe mental illness: systematic review and meta-analysis. Frontiers in Endocrinology, 12, 769309.

Beck, A. T., Steer, R. A., & Brown, G. (1996). Beck depression inventory–II. San Antonio, 78(2), 490-498

Boland, R., Verdiun, M. and Ruiz, P. (2022). Kaplan & Sadock's synopsis of psychiatry, 12th edition. Wolters Kluwer.

Carrà, G., Bartoli, F., Carretta, D., Crocamo, C., Bozzetti, A., Clerici, M., & Bebbington, P. E. (2014). The prevalence of metabolic syndrome in people with severe mental illness: a mediation analysis. Social Psychiatry and Psychiatric Epidemiology, 49, 1739-1746.

Eder, J., Glocker, C., Barton, B., Sarisik, E., Popovic, D., Lämmermann, J., ... & Musil, R. (2024). Who is at risk for weight gain after weight-gain associated treatment with antipsychotics, antidepressants, and mood stabilizers: A machine learning approach. *Acta Psychiatrica Scandinavica*.

Ferdowsy, F., Rahi, K. S. A., Jabiullah, M. I., & Habib, M. T. (2021). A machine learning approach for obesity risk prediction. Current Research in Behavioral Sciences, 2, 100053.

Gill, H., Gill, B., El-Halabi, S., Chen-Li, D., Lipsitz, O., Rosenblat, J. D., . . . Majeed, A. (2020). Antidepressant medications and weight change: a narrative review. Obesity, 28(11), 2064-2072.

Hirschfeld, R. M., Williams, J. B., Spitzer, R. L., Calabrese, J. R., Flynn, L., Keck Jr, P. E., . . . Rapport, D. J. (2000). Development and validation of a screening instrument for bipolar spectrum disorder: the Mood Disorder Questionnaire. American journal of psychiatry, 157(11), 1873-1875.

JON, D.-I., YOON, B.-H., JUNG, H.-Y., HA, K.-S., SHIN, Y.-C., & BAHK, W.-M. (2005). A validation study of the Korean version Mood Disorder Questionnaire (K-MDQ). Journal of Korean Neuropsychiatric Association, 44(5), 583-590.

Kay, S. R., Fiszbein, A., & Opler, L. A. (1987). The positive and negative syndrome scale (PANSS) for schizophrenia. Schizophrenia bulletin, 13(2), 261-276.

Kim, B.-Y., Kang, S. M., Kang, J.-H., Kang, S. Y., Kim, K. K., Kim, K.-B., . . . Kim, J.-H. (2021). 2020 Korean Society for the Study of Obesity guidelines for the management of obesity in Korea. Journal of obesity & metabolic syndrome, 30(2), 81-92.

Kim, K.-K., Haam, J.-H., Kim, B. T., Kim, E. M., Park, J. H., Rhee, S. Y., . . . Koo, H. Y. (2023). Evaluation and Treatment of Obesity and Its Comorbidities: 2022 Update of Clinical Practice Guidelines for Obesity by the Korean Society for the Study of Obesity. Journal of obesity & metabolic syndrome, 32(1), 1-24.

Mazereel, V., Detraux, J., Vancampfort, D., Van Winkel, R., & De Hert, M. (2020). Impact of psychotropic medication effects on obesity and the metabolic syndrome in people with serious mental illness. Frontiers in Endocrinology, 11, 573479.

Nicklas, J. M., Huskey, K. W., Davis, R. B., & Wee, C. C. (2012). Successful weight loss among obese US adults. American journal of preventive medicine, 42(5), 481-485.

Sabé, M., Pallis, K., Solmi, M., Crippa, A., Sentissi, O., & Kaiser, S. (2023). Comparative Effects of 11 Antipsychotics on Weight Gain and Metabolic Function in Patients With Acute Schizophrenia: A Dose-Response Meta-Analysis. The Journal of Clinical Psychiatry, 84(2), 45463.

Serretti, A., Mandelli, L., & Laura, M. (2010). Antidepressants and body weight: a comprehensive review and meta-analysis. The Journal of Clinical Psychiatry, 71(10), 1259-1272.

Stogios, N., Humber, B., Agarwal, S. M., & Hahn, M. (2023). Antipsychotic-Induced Weight Gain in Severe Mental Illness: Risk Factors and Special Considerations. Current Psychiatry Reports, 25(11), 707-721.

Zheng, P., Yu, Z., Mo, L., Zhang, Y., Lyu, C., Yu, Y., ... & Li, Y. (2022). An individualized medication model of sodium valproate for patients with bipolar disorder based on machine learning and deep learning techniques. *Frontiers in Pharmacology*, *13*, 890221.

강지현, & 김경곤. (2024). 일차의료기관에서의 비만 클리닉 진료 지침. *대한의사협회지*, *67*(4), 240-255.

김성훈, 김경민, 윤보현, 정하란, 정유란, 윤현주, . . . 시영화. (2021). 대사증후군을 가진 중증정신질환자의 장기 추적관찰: 10 년 추적연구 (2011~ 2020). 생물치료정신의학, 27(1), 12-24.

대한비만학회. (2022). 비만진료지침 8 판 요약본

손장원. (2022). 비만 약물치료의 최신지견. *Journal of Korean Diabetes*, *23*(2). 113-127.

이자해. (2014). 비만 환자에서 장기간의 체중 감량 유지 관련 요인. 울산대학교 대학원, 울산. (국내석사학위논문)

이중서, 안용민, 신현균, 안석균, 주연호, 김승현, . . . 이지연. (2001). 한국판 양성 및 음성증후군 척도 (Positive and Negative Syndrome Scale)의 신뢰도와 타당도. 신경정신의학, 40(6), 1090-1105.

질병관리청. (2023 년 12 월 8 일). 국민건강영양조사 제 9 기 1 차년도(2022). https://www.kdca.go.kr/board/board.es?mid=a20501010000&bid=0015&act=view&list_no=724014

한국건강증진개발원. (2021). 국민건강증진을 위한 비만 통계자료집(2016-2020). (사업-02-2021-019-15).
